# Native forest metacommunity structures in Uruguay shaped by novel land‐use types in their surroundings

**DOI:** 10.1002/ece3.8700

**Published:** 2022-03-06

**Authors:** Leonardo R. Ramírez, Ina Säumel

**Affiliations:** ^1^ Integrative Research Institute THESys Transformation of Human‐Environment‐Systems Humboldt‐Universität zu Berlin Berlin Germany

**Keywords:** Campos region, Clementsian structure, community dissimilarity, dispersal limitation, landscape configuration, nestedness, spatial heterogeneity, species turnover

## Abstract

We explore the effect of land‐use change from extensively used grasslands to intensified silvi‐ and agricultural monocultures on metacommunity structure of native forests in Uruguay. We integrated methods from metacommunity studies, remote sensing, and landscape ecology to explore how woody species distribution was influenced by land‐use change from local to regional scale. We recorded richness and composition of adult and juvenile woody species from 32 native forests, created land‐use maps from satellite image to calculate spatial metrics at landscape, class, and patch levels. We also analyzed the influence of land use pattern, climate, topography, and geographic distance between sites (*d*) on metacommunity, and created maps to visualize species richness and (dis)similarity between communities across the country. Woody species communities were distributed in a discrete pattern across Uruguay. Precipitation and temperature seasonality shaped species distribution pattern. Species richness and community dissimilarity increased from West to East. Latitude did not influence these patterns. Number of patches, landscape complexity, and interspersion and juxtaposition indexes determine woody species distribution at landscape level. Increasing areas covered by crops and timber plantation reduced species richness and increased community dissimilarity. The spatial metrics of native forest fragments at patch level did not influence metacommunity structure, species richness, and community dissimilarity. In conclusion, Uruguayan native forests display a high range of dissimilarity. Pressure of neighborhood land uses was the predominant factor for species assemblages. Conserving landscape structures that assure connectivity within and among native forest patches is crucial. On sites with rare target species, the creation of alliances between governmental institution and landowner complemented by incentives for biodiversity conservation provides opportunities to advance in species protection focused on those less tolerant to land‐use change.

## Graphical abstract

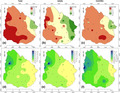

## 1 INTRODUCTION

**FIGURE 1:**
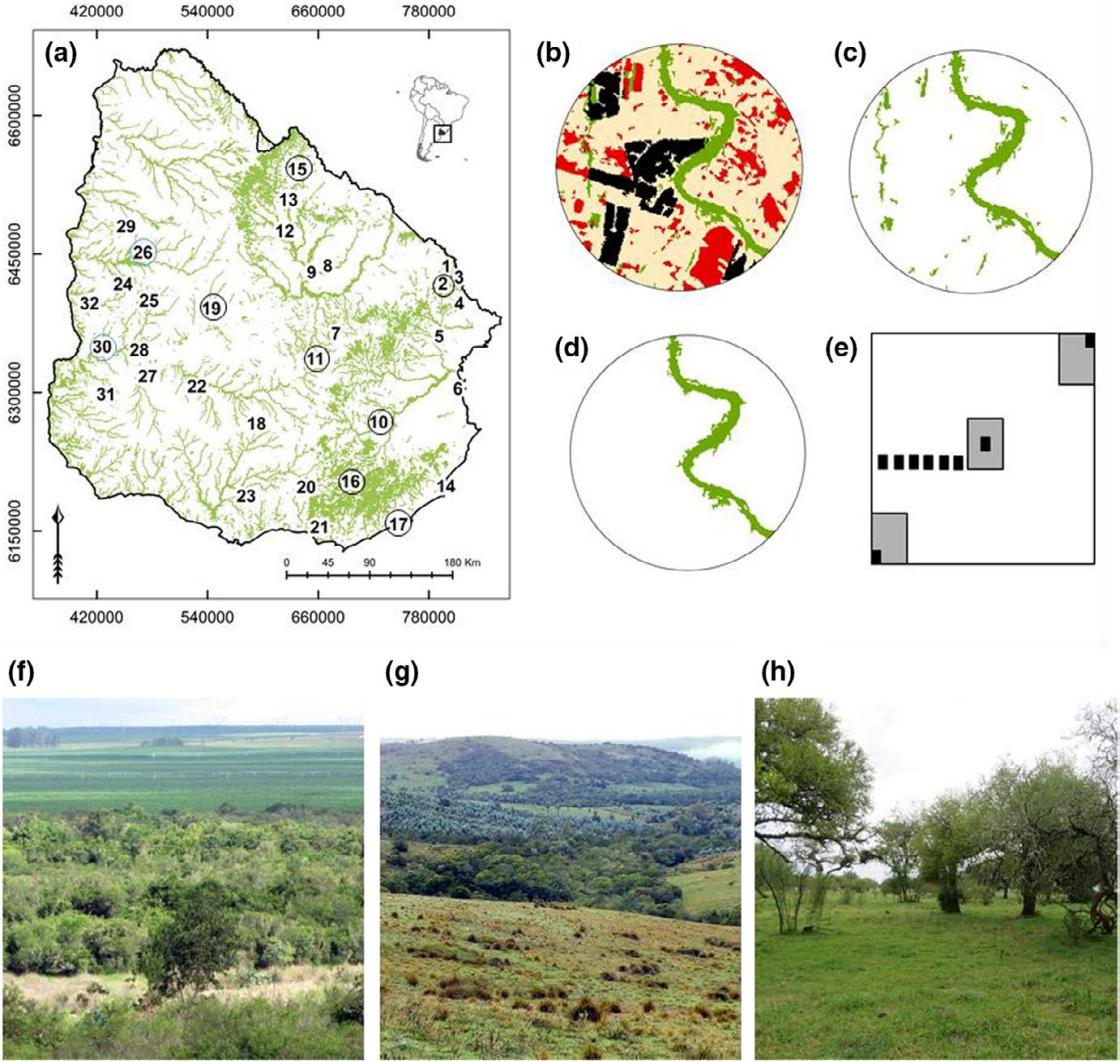
Study area and land use within a buffer of 3 km from the central point of each of plot (*N* = 32). (a) Distribution of native forests and permanent monitoring plots across Uruguay; no‐circle = riverine forest, black circle = hill forest, and blue circle = park forest, (b) land‐use map with different patches and classes, (c) native forest patches distribution within landscape, (d) native forest patch where permanent plot was established, and (e) permanent plot (100 × 100 m) and subplots. For (a), (b), (c), and (d), colors represent different land‐use types: green = native forest, black = timber plantation, red = crops, and beige = natural grassland. For (e), gray square = subplots woody adults (10 × 20 m), black square = juvenile subplots (3 × 3m). The photographs show (f) riverine forests with crops in background, (g) hill forests at hillsides with *Eucalyptus* plantations, and (h) park forests in the transition zones between riverine forests and extensively used grasslands

Land‐use changes across the world threaten biodiversity, reduce habitat connectivity, and the provision of ecosystem services (Cardinale et al., 2012; Foley et al., 2005). In Uruguay, global markets and local governmental policies have driven land‐use shifts, from extensively used grasslands, the so called “Campo natural” to high yield plantations of silvi‐ and agriculture (Alvarez et al., 2015). Within the dominant matrix of temperate grasslands, small spatial patches of native forests cover around six percent of Uruguay (Figure 1a; Alvarez et al., 2015). Although land‐use change occurs mainly on the expense of grassland, cross boundary effects of neighboring land use on native forests have been demonstrated (Ramírez & Säumel, 2022) and have to be considered to reduce the trade‐offs between biodiversity conservation and economic profit.

Uruguayan forests have been used for extraction of timber and firewood at least since the European colonization. They have been classified according to their physiognomy and topographic localization into riverine forests, park forests in the transition zones between riverine forest and extensively used grasslands, and some hill forests at hillsides, and gulches (Figure 1a,f–h; Brussa & Grela, 2007; Haretche et al., 2012). Few studies that exist on them propose that woody species composition responds to geology‐ (Gautreau & Lezama, 2009) or to topography‐related water gradients (Traversa‐Tejero & Alejano‐Monge, 2013). There are no studies on the effects of current land‐use change on metacommunity structures of Uruguayan native forest. Recent studies on native forest from Southeastern Brazil indicated synergic effects between environment and human activities on woody species composition at different spatial scales (da Silva & Rossa‐Feres, 2017; Marcilio‐Silva et al., 2017; Neves et al., 2017; Oliviera‐Filho et al., 2015). Changes in environmental conditions drive local endemism (Neves et al., 2017) and introduction of exotic species (Zwiener et al., 2018) at local scale, and homogenization of species composition at regional and landscape scale (Oliviera‐Filho et al., 2015; Zwiener et al., 2018).

Here, we analyze metacommunity structures of native forests across Uruguay to disentangle regional pattern of biodiversity (Leibold et al., 2004). The metacommunity concept defines interconnected ecological communities depending on the flow and exchange of species and responding to spatial heterogeneity (Leibold & Chase, 2018). Metacommunities are characterized by distribution pattern of species shared between sites, by species turnover between sites, and how boundaries of different species are clustered (Leibold & Mikkelson, 2002; Presley et al., 2010). Exchange of species between communities depends on the intrinsic characteristics of species such as dispersal or life‐history traits, the distance between habitats, and the availability of ecological niches (MacArthur & Wilson, 1967). Different land uses generate limitations for species dispersal between patches, form, and shape environmental filters, that in turn influence species establishment as well as inter‐ and intraspecies competition (Chase & Leibold, 2003; Tilman, 1982).

The metacommunity concept has been successfully applied to analyze responses of communities to habitat loss and fragmentation (de la Sancha et al., 2014), and to explain species distribution patterns of woody plants (Marcilio‐Silva et al., 2017). Here, we explore impacts of land‐use change on metacommunity structures of Uruguayan native forest. We focus on the influence of landscape features and spatial metrics of the changing landscape on woody communities in order to inform land management and biodiversity conservation. We specifically address the following questions: (i) how native forest communities are structured across Uruguay, (ii) which environmental factors are underlying to the distribution of woody species, and (iii) how land‐use change in the South American grassland biome impacts on metacommunity structure of native forest.

## 2 MATERIALS AND METHODS

We used a stratified randomized design. In a first step, we used a randomized design for the selection of monitoring sites across the country. Second, we contacted the potential landowners to explore their willingness to establish long‐term monitoring sites. In total, we established 32 plots (100 × 100 m) in different native forest fragments across Uruguay (Figure 1).

In two vegetations periods (from December 2015 to April 2016 and from October 2016 to January 2017), we recorded all woody species in two size‐classes based on diameter at breast height (dbh). We take the size‐classes as a noninvasive proxy measure for tree age to differentiate in: adults (dbh ≥ 5 cm) recorded in 3 plots of 10 × 20 m and juveniles (dbh < 5 cm) recorded in 9 plots of 3 × 3 m (Figure 1e). We used the dbh of 5 cm as limit between juveniles and adult individuals based on regional literature (Alves et al., 2010; Ribeiro et al., 2011). The woody species in the local forests comprise also multistem species, that are not easily categorized in trees or shrubs. Depending on the local condition these species have more a growth habit of a shrub or more of a tree (e.g., *Blepharocalyx salicifolius*, *Eugenia uniflora*, *or Maytenus ilicifolia*). Classification in shrubs, trees, and those species that can have both growth habits are indicated in Table S1. All names of species identified were updated using the online database from The Plant List v.1.1 (2013).

We created three presence/absence matrices on the basis of age‐classes and all woody species (juvenile and adult species). We further categorized species according to dispersal syndrome (zoochory, anemochory, and autochory), origin (native and exotic) according to Uruguayan conservation priority (priority, nonpriority, and nonevaluated; see Table S1) to facilitate information for land management and conservation measures.

In order to determine the most common and rare species, we calculated frequencies: absolute frequency as the number of times that one species was registered across all sites (i.e., maximum frequency was 32 and minimum frequency was 1); relative frequency as percentage of presence across sites (i.e., absolute frequency divided by total sites); and cumulative relative frequency as absolute frequency of one species divided by sum of absolute frequencies for all species, multiplied by 100 to transform it into a percentage.

We built matrices with species and sites (rows and columns in the matrix) to analyze the proximity between sites with similar species composition and species with similar distribution (Leibold & Mikkelson, 2002; Presley et al., 2010). This technique shows indirectly whether species distributions are ordered in response to environmental gradients (Gauch et al., 1977).

The metacommunity structure was described by different elements (Leibold & Mikkelson, 2002; Presley et al., 2010): coherence (i.e., number of interruptions in species distribution across the sites), species turnover (i.e., number of species replacements between two sites), and boundary clumping (i.e., boundaries in species composition across two or more sites based on the Morisita overlap index; see detail in Table S2). The EMS were calculated with Matlab (The Mathworks Inc., Natick, MA, USA), using a script developed by Presley and Higgins (n.d).

We determined the elements of metacommunity structure (EMS) for matrix of adult individuals, juvenile individuals of the regenerating layer, and total species (sum of adult and juvenile woody species). The models for matrix ordination were set by reciprocal averaging (Table S3; Gauch et al., 1977), the null model with fixed species richness per site, and equiprobable species occurrence (random 0). The models ran with 1000 iterations and extractions of the scores from the first axis of ordination based on reciprocal averaging. We used the score from the first axis to correlate with environmental variables and landscape metrics (see Tables 2 and 3; Table S4).

We created species distribution richness‐range maps and composition similarity‐range maps by multivariate interpolation using inverse distance‐weighted technique with ArcGIS v.10.3.1 for Desktop (ESRI). We calculated environmental variables by extracting bioclimatic, geographic, and topographic data from available spatial databases for each permanent plot, using the coordinates of the central point each permanent plot to extract the information (Table S5). The bioclimatic variables were extracted from the WorldClim v.2 database (Fick & Hijmans, 2017) at spatial resolution of 30 s. The geographic data were based on latitude and longitude of the central point of each permanent plot based on UTM coordinate system. The topographic data were extracted from institutional digital elevation model (MVOTMA 2017), and elevation and slope in percentage was calculated (%, Table S5).

We classified land use from Landsat 8 OLI satellite image for the year 2017 (U.S. Geological Survey, 2017) in a buffer zone of 3km from central point of each permanent plot, processing atmospheric and geometric correction by Landsat image using Matlab (The Mathworks Inc.). We combined two techniques of classification: we first used supervised classification using ground control points collected in a field across different land uses to capture signature spectral of each land‐use type, then used tree classification technics based on signature spectral of each land‐use type with ENVI v.5.3 (Exelis Visual Information Solutions). The land‐use maps were set to six land‐use types (i.e., native forest, grassland, timber plantation, agriculture, water body, and urban areas). Due to the small area covered by water bodies and urban areas, these land uses were not considered in the analysis.

We used the land‐use maps to calculate spatial metrics based on landscape composition (i.e., diversity and abundance of patch types) and landscape configuration (i.e., spatial features and arrangement of patches and classes within the landscape; Table S2). Composition and configuration of landscape was calculated in three levels: patch, class, and landscape (Figure 1b–e). A patch is a homogeneous area within a landscape with specific biotic and abiotic features, and a class is a set of patches with the same features (i.e., a specific land‐use type; McGarigal et al., 2012). All spatial metrics were calculated using Fragstat v.4 (McGarigal et al., 2012).

### 2.1 Data analysis

We used Pearson correlation analysis using Past 3.16 (Hammer et al., 2001). To evaluate whether metacommunity structure (i.e., adult, juveniles, and both age‐classes together) responded to climatic, geographic location and topographic variables, and landscape metrics, we determined Pearson coefficient (*r*) based on lineal association between the scores of the first axis of ordination generated by reciprocal averaging with each environmental variable and landscape metric. We also explored relationships between species richness and landscape metric by Pearson correlation analysis.

We created a matrix distance‐similarity to determine whether geographic distance influenced the similarity of species composition between sites. The distance between sites was calculated using ArcGIS v.10.3.1 for Desktop (ESRI), and composition similarity was based on Jaccard Index (J) using Past 3.16 (Hammer et al., 2001). The matrix distance‐similarity was calculated to both age‐classes together. We created species distribution richness‐range maps and composition similarity‐range maps by multivariate interpolation using inverse distance‐weighted technique with ArcGIS v.10.3.1 for Desktop (ESRI).

We performed a Mantel test and a partial Mantel test to examine the association between community dissimilarity with environmental variables and landscape metrics. Dissimilarity was calculated using the Jaccard index. For the partial Mantel test, geographic distance between plots was included as a third matrix. Mantel test and partial Mantel test were performed using the Vegan package (Oksanen et al., 2020) implemented in R (R Core Team., 2020). We performed 9999 permutations for the community distance matrix and evaluated with the Pearson coefficient at the significance level of *p* < .05 (Oksanen et al., 2020).

We further calculated a linear regression that best fitted our data to determine if species composition across sites to predict the influence of land‐use pattern, and selected the environmental factors with higher correlation to species richness as the independent variable. The best linear regression model was selected based on Akaike Information Criterion (AIC; Akaike, 1974).

## 3 RESULTS

### 3.1 Diverse species composition of native forests

**FIGURE 2:**
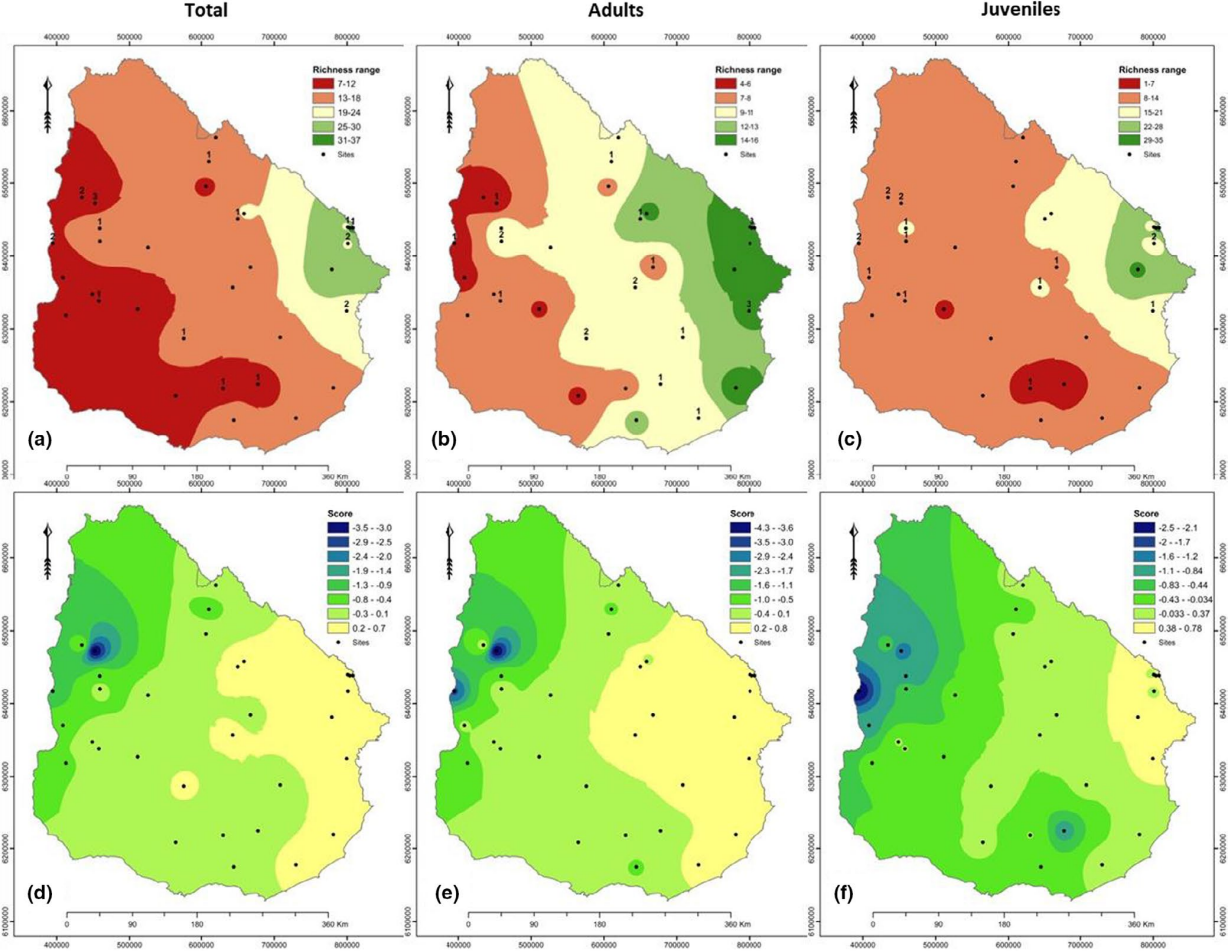
Maps of woody species richness‐range (a–c) and similarity‐range based on Jaccard index (d–f) across Uruguay for individuals of all age‐classes (a, d), for adults (b, e), and for juveniles (c, f). The total number of woody species per native forest fragment was, for adults, between 4 and 16 (mean = 10.1; SD = 3.4); for juveniles, between 1 and 35 (mean = 13.4; SD = 7.2); and for both age‐classes together, between 7 and 37 (mean = 16.3; SD = 6.9). Riverine forests harbor between 7 and 34 (mean = 16.4; SD = 6.6), and hill forests between 10 and 37 species (mean = 17.7; SD = 8.8)

**FIGURE 3:**
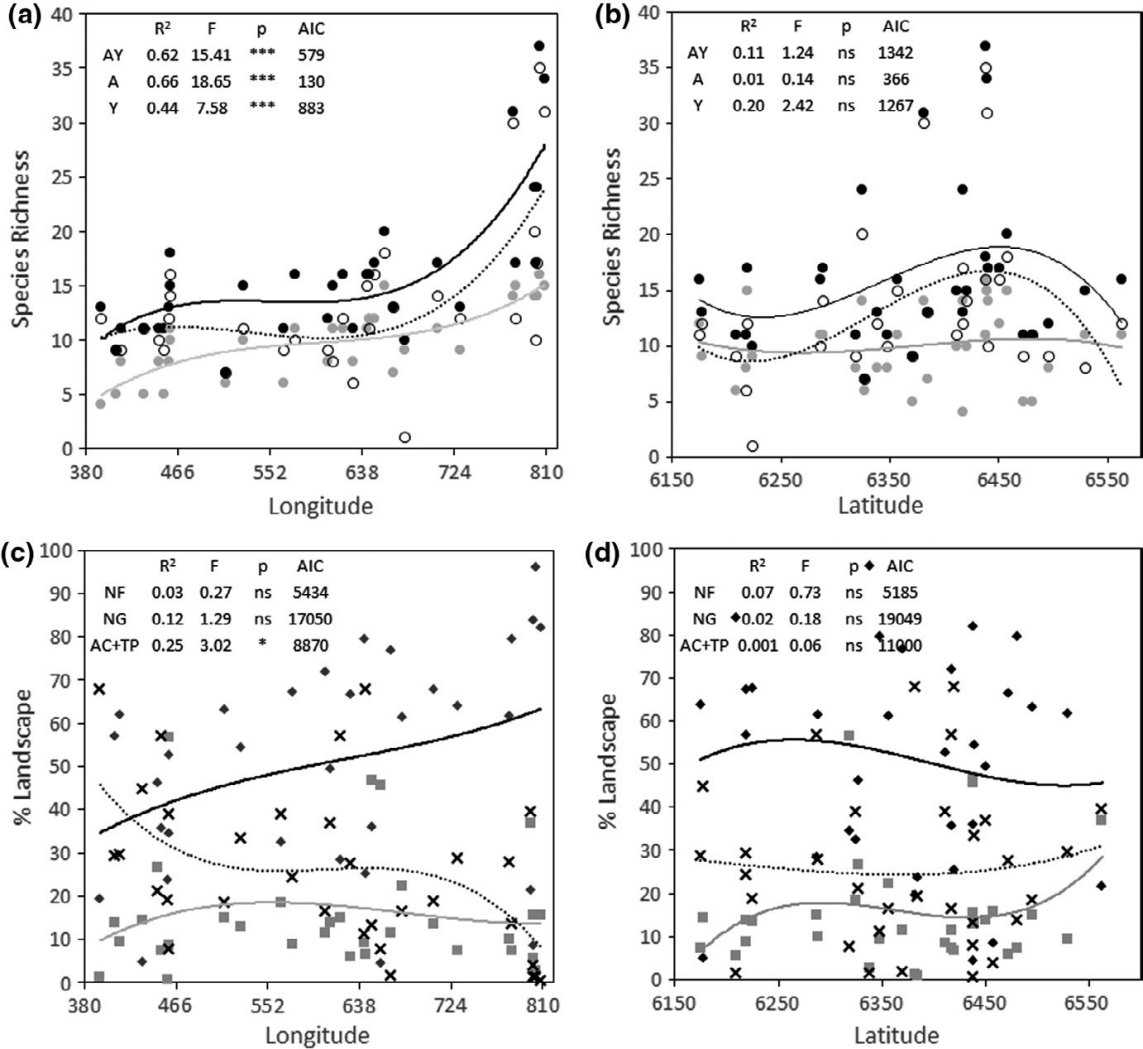
Linear regression for (a) longitude versus species richness, (b) latitude versus richness, (c) longitude versus percentage by land‐use type, and (d) latitude versus percentage by land‐use type. For (a) and (b); AY = all woody species (black circle and black line), A = adult (gray circle and grey line), Y = juveniles (white circle and dashed line). For (c) and (d); NF = percentage of native forest (gray square and gray line), NG percentage of natural grassland (black diamond and black line), AC+TP = percentage crops and timber plantation together (black cruxes and dashed line). All regressions are polynomials of the third order. Longitude and latitude are given in UTM/1000. We found the highest species richness between coordinates eastern longitudes of 750,000 and 810,000 and between southern latitudes of 6,300,000 and 6,440,000 (UTM coordinates)

In total, we registered 41 families, 77 genera, and 101 woody species across native forests of Uruguay (Table S1). Four families (Myrtaceae, Fabaceae, Anacardiaceae, and Salicaceae) represented 83% of total richness. We found the same species with higher relative frequency for adults, juveniles, and individuals from both age‐classes (i.e., *Allophylus edulis*, *Scutia buxifolia*, and *Blepharocalyx salicifolius*). Of all species, 35% occurred only once across all sites. Species richness increased asymptotically for all age‐classes from the Western to Eastern Uruguay (Figure 2a–c). The best fitting regression were polynomials of the third order (AIC, Figure 3a). The influence of latitude on woody species richness was not significant (Figure 3b).

Of all recorded species, 93% are native, except seven exotics (Table S1). More than 70% of all species are classified as zoochore (*N* = 72). Nine species are anemochore and eight autochore (Table S1). Eight species have conservation priority status (Soutullo et al., 2013; Table S2). We recorded adults of thirteen native species without any presence of juvenile individuals, among them *Butia odorata*, which is categorized as high priority for conservation (Table S1). All occur with low frequency, except the hemiparasitic mistletoe *Tripodanthus acutifolius*.

Of the species, 26 were recorded only in the regeneration layer but not among adults. All are native to the region, except the South‐East Asian *Melia azedarach*, the Chinese *Poncirus trifoliata*, and the European *Pyracantha coccinea* (Table S1). Most frequent species are the climbing *Celtis iguanaea*, *Smilax campestris*, and the shrubby *Heimia salicifolia*. Five of the native species that only occurred in the regeneration layer have conservation priority (i.e., *Casearia decandra*, *Actinostemon concolor*, *Maytenus dasyclados*, *Phytolacca americana*, and *Xylosma schroederi*). In addition, we recorded 27 species only at one site as adults, 17 species only at one site in the regeneration layer, and 9 species only at one site but as adults and juvenile (Table S1).

### 3.2 Metacommunity structure of native forests

Across all forest types and in riverine forests alone, the adults, juveniles, and individuals from both age‐classes together displayed a Clementsian distribution (Table 1). The analysis of elements of metacommunity structure revealed a positive coherence (i.e., less embedded absences than expected by chance), a positive species turnover (i.e., more replacements than expected by chance), and a significant boundary clumping with a Morisita Index higher than one.

**TABLE 1:** Coherence, species turnover, boundary clumping, and idealized pattern of metacommunity for adult woody species (dbh ≥ 5 cm), juvenile (dbh < 5 cm) woody species, and both age‐classes together (all) from different native forests across Uruguay

Forest types	Age‐classes	Coherence				Species turnover				Boundary clumping		Idealized pattern of species distribution
		Abs	*p*	Mean	SD	Re	*p*	Mean	SD	MI	*p*	
All (*n* = 32)	Adults	942	**<.0001**	1279	50.7	31435	.**0028**	24318	2381	1.19	.**0053**	Clementsian
	Juveniles	1015	**<.0001**	1629	65.9	47161	.**0002**	34456	3384	1.46	**<.0001**	Clementsian
	All	1334	**<.0001**	1991	59.1	64819	**<.0001**	45927	4415	1.28	**<.0001**	Clementsian
Riverine forests (*n* = 23)	Adults	389	**<.0001**	692.45	36.00	14540	.**0091**	11719.36	1081.80	1.37	**<.0001**	Clementsian
	Juveniles	652	**<.0001**	943.78	42.60	20040	**<.0001**	13857.13	1490.22	1.45	**<.0001**	Clementsian
	All	737	**<.0001**	1149.85	44.15	25280	.**0002**	18538.21	1791.47	1.28	**<.0001**	Clementsian
Hill forests (*n* = 7)	Adults	60	.4969	66.57	9.67	961	.**0136**	781.36	72.82	0.93	.1734	Random
	Juveniles	42	.**0165**	87.99	19.17	887	.**0002**	1403.84	140.90	1.04	.2408	Nested ‐stochastic species loss
	All	82	.**0055**	119.48	13.50	1305	.8389	1332.45	134.98	1.05	.2031	Quasi‐nested Stochastic species loss

Note

Mean and SD were calculated from 1000 iterations of null matrices (based on Leibold & Mikkelson, 2002; Presley et al., 2010).

Abbreviations: Abs, number of embedded species; MI, overlap Morisita Index; *p*, *p*‐value; Re, number of replacements; SD, standard deviation.

We observed different patterns in hill forests (“Serrano” forests or “Quebradas”): juveniles and individuals from both age‐classes together showed a positive coherence and a (quasi) nested distribution with stochastic species loss (Table 1). Taxa found in species‐poorer sites were subsets of those found in species‐richer sites. In contrast, metacommunity structure of adult species followed a random pattern.

### 3.3 Response of metacommunities to environmental gradients

Longitude was positively associated with metacommunity structure and species richness and community dissimilarity increased from west to east (Table 2, Table S6). In contrast, latitude, elevation and slope were not correlated with either metacommunity structures, species richness, and community dissimilarity (Table 2, Tables S6 and S7). The metacommunity structures were related to seasonality of temperature. Species richness of woody species was positively related to the mean temperature of the driest quarter of the year, and negatively related to the mean temperature of wettest quarter and overall temperature seasonality. Community dissimilarity was also positively related to the mean temperature of the driest quarter of the year (Tables S6 and S7). Species richness of juveniles in the native forests and individuals from all age classes were also positively related to the mean temperature of the coldest quarter of the year (Table S4).

**TABLE 2:** Pearson's correlation between the metacommunity structure based on the first axis of ordination (reciprocal averaging) and species diversity of woody species arrangement and richness (all species, adult, and juvenile individuals) versus longitude/latitude, meter above sea level, slope, and bioclimatic variables

Variable	First axis ordination						Species richness					
	All woody species		Adults		Juveniles		All woody species		Adults		Juveniles	
	Pearson	*p*	Pearson	*p*	Pearson	*p*	Pearson	*p*	Pearson	*p*	Pearson	*p*
Longitude (UTM)	**0.59**	.**0004**	**0.56**	.**0009**	**0.70**	**<.0001**	**0.67**	**<.0001**	**0.79**	**<.0001**	**0.51**	.**0031**
Latitude (UTM)	−0.22	.2278	−0.15	.4051	−0.07	.7054	0.23	.2075	0.07	.6872	0.27	.1361
Meter above sea level	0.04	.8135	0.14	.4387	0.11	.5508	0.04	.8135	0.14	.4387	0.11	.5508
Slope (%)	0.13	.4884	0.08	.6704	0.11	.5332	0.13	.4884	0.08	.6704	0.11	.5332
Annual mean temperature	−0.30	.1004	−0.26	.1542	−0.17	.3553	0.12	.5214	−0.10	.5742	0.23	.2006
Temperature seasonality	**−0.40**	.**0252**	**−0.36**	.**0437**	**−0.45**	.**0091**	**−0.53**	.**0017**	**−0.66**	**<.0001**	**−0.36**	.**0406**
Annual precipitation	0.06	.7339	0.16	.3789	0.28	.1250	0.34	.0576	**0.37**	.**0382**	0.22	.2158
Precipitation seasonality	**−0.59**	.**0004**	**−0.59**	.**0004**	**−0.66**	**<.0001**	−0.27	.1424	**−0.52**	.**0021**	−0.12	.5146

Note

*p*‐values in bold indicate significance with *p* < .05. For more information on bioclimatic variables see Table S6.

The metacommunity structure, species richness, and community dissimilarity were also linked to precipitation variables (Table 2, Tables S4, S6, S7). For metacommunity structure there was a positive correlation with the precipitation during the coldest quarter and the driest quarter of the year, and a negative correlation with the precipitation seasonality (Table 2). The precipitation of the wettest quarter of the year was negatively correlated with the woody species community structure of both age‐classes together. The community dissimilarity increased with precipitations where the correlation of precipitation of coldest quarter was higher (Tables S6 and S7).

### 3.4 Response of metacommunity structure to landscape

The metacommunity structure of all woody species was negatively correlated with the number of patches at landscape scale (Table 3). The metacommunity structure of juveniles was negatively correlated with landscape shape index and positively related to aggregation index.

**TABLE 3:** Pearson's correlation between the metacommunity structure based on the first axis of ordination (reciprocal averaging) and the diversity of woody species arrangement and richness (all species, adult, and juvenile) versus landscape metrics

Level	Indices	First axis ordination						Species richness					
		All		Adult species		Juvenile species		All		Adults species		Juvenile species	
		Pearson	*p*	Pearson	*p*	Pearson	*p*	Pearson	*p*	Pearson	*p*	Pearson	*p*
Landscape	Number of patches	**−0.36**	.**0453**	−0.33	.0694	−0.33	.0612	−0.32	.0745	−0.30	.0928	−0.30	.0957
	Landscape shape index	−0.34	.0593	−0.33	.0665	**−0.41**	.**0195**	**−0.48**	.**0058**	**−0.48**	.**0051**	**−0.42**	.**0176**
	Shannon's evenness index	−0.28	.1144	−0.22	.2263	−0.34	.0561	**−0.61**	.**0002**	**−0.52**	.**0021**	**−0.55**	.**0011**
	Aggregation index	0.34	.0602	0.33	.0670	**0.41**	.**0198**	**0.47**	.**0070**	**−0.48**	.**0059**	**0.41**	.**0197**
	Percentage of cover by native forest	0.13	.4668	0.17	.3443	0.07	.7118	0.08	.6493	0.23	.1970	0.10	.5874
	Percentage of cover by natural grassland	0.29	.1118	0.33	.0645	0.29	.1100	0.18	.3159	0.20	.2801	0.15	.4015
	Percentage of cover by crops	−0.15	.4223	**−0.36**	.**0449**	−0.35	.0539	−0.16	.4011	−0.23	.2058	−0.18	.3221
	Percentage of cover by timber plantation	**−0.55**	.**0028**	**−0.50**	.**0073**	−0.38	.0505	−0.26	.1872	**−0.43**	.**0244**	−0.22	.2712
	Percentage of cover by sum of timber plantation and crops	**−0.55**	.**0012**	**−0.61**	.**0002**	**−0.50**	.**0036**	−0.35	.0526	**−0.46**	.**0085**	−0.32	.0785
	Number of native forest patches	−0.32	.0734	−0.18	.3265	−0.24	.1851	−0.15	.3981	−0.19	.2875	−0.17	.3423
	Interspersion and juxtaposition index for native forest patches	−0.34	.0552	**−0.37**	.**0346**	**−0.38**	.**0319**	**−0.37**	.**0366**	**−0.42**	.**0154**	−0.32	.0764
	Mean Euclidean nearest neighbor distance of native forest patches	0.10	.5813	−0.04	.8483	0.17	.3591	0.06	.7298	0.10	.5754	0.01	.965
Patch	Total Area	0.05	.7701	0.08	.6683	0.01	.9753	0.09	.6397	0.23	.2062	0.13	.4874
	Perimeter‐Area Ratio	−0.18	.3284	−0.19	.3044	−0.16	.3898	0.05	.7897	−0.11	.555	0.03	.8798
	Shape Index	0.18	.3160	0.25	.1614	0.13	.4692	0.07	.7159	0.07	.7156	0.07	.6962

Note

*p*‐values in bold indicate significance with *p* < .05.

The general metacommunity structure of woody species was negatively correlated to the cumulative percentage of the landscape covered by timber plantation and crops. The percentage of cover by timber plantation was negatively associated with metacommunity structure of adults and both age‐classes together (Table 3). The percentage of cover by crops was only correlated with the metacommunity structure of adult woody species. The percentage of a landscape covered by native forest and grassland was not related to the metacommunity structure of woody species.

Based on the metrics related to native forest fragments within the landscape (Figure 1c), the interspersion and juxtaposition index is negatively related to the metacommunity structure of adults and juveniles. Neither the number of patches of native forest nor the mean Euclidian nearest neighbor distance between native forest patches was associated with the metacommunity structure of adult woody species, juveniles, and species of both age‐classes together. At native forest patch level (Figure 1d), the total area of fragments, perimeter‐area ratio, and shape index were not associated with the arrangement of metacommunities.

Community dissimilarity was influenced by landscape metrics at all levels (i.e., landscape, class and patch). Productive land uses (i.e., timber plantations and cropland, Tables S8 and S9) determine differences in the composition of woody communities.

### 3.5 Species richness and landscape metrics

At landscape scale, species richness of woody species decreased with increasing Shannon's evenness index and landscape shape index. Species richness of all woody species increased with increasing aggregation index. Juvenile woody species richness increased with increasing aggregation index, while adult species richness decreased with increasing aggregation index (Table 3).

Species richness of adults decreased and the community dissimilarity increased with increasing cover by timber plantation and by both novel land‐use types together (crops and timber plantation; Table 3, Tables S8 and S9). The proportion of the landscape covered by natural grassland and native forest was not correlated with species richness nor community dissimilarity in our native forest plots. Species richness of adults, and both adults and juveniles together decreased with increasing interspersion and juxtaposition index. At patch level (native forest fragments), the total area, perimeter area ratio, and shape index were not correlated with species richness nor community dissimilarity of all woody species, adults, and juveniles (Table 3, Tables S8 and S9).

### 3.6 Similarity and geographic distance between forest communities

Geographic distances between all sites ranged from 35 km to 415 km. The highest similarities in species composition between native forest communities (Jaccard Index (*J*) ≥ 0.70) were recorded at the geographically near sites 8 and 9 (*d* = 11 km; *J* = 0.76) and at the geographically distant sites 12 and 28 (*d* = 218 km; *J* = 0.77). Medium values of similarity (0.50 ≤ *J* < 0.70) were found between geographically near sites 1 and 2 (*d* = 4 km; *J* = 0.54) and between geographically distant sites 12 and 31 (*d* = 262 km; *J* = 0.53). The site with the highest dissimilarities compared to other sites (*J* ≤ 0.10) was site 26 (Table S10). Woody species composition at this site was markedly different from more than 60% (*n* = 19) of all sites.

When geographic distance was considered separately the significant difference in woody species composition was recorded between sites 2 and 22 (*d* = 313 km; *J* = 0.073), between sites 11 and 22 (*d* = 135 km; *J* = 0.095), between sites 2 and 29 (*d* = 372 km; *J* = 0.091), between sites 7 and 30 (d = 259km; J = 0.100), and between sites 1 and 32 (d = 414km; J = 0.093; see Table S10 and Figure 3).

## 4 DISCUSSION

To our knowledge, this is the first study to apply the framework of metacommunity structure combined with environmental drivers and landscape metrics to explore the distribution of woody species in native forests across Uruguay. As the distribution of woody species followed a Clementsian pattern, the species of the forest communities are distributed in a discrete pattern across the country (Table 1). The high level of coherence indicates that species and communities are ordered following the environmental gradient. The communities replace each other as a group based on species turnover and the distribution of species’ range (Table 1; Leibold & Mikkelson, 2002; Presley et al., 2010). This pattern is influenced by the high number of species that we recorded only at one site. Thus, each community harbors endemic species, and 43% of all recorded species are recorded only at one site (Table S1).

Similar metacommunity arrangements were strongly related to the longitudinal ordination and to short geographic distances (Figure 3), except those with more than 200 km between very similar species assemblages (i.e., site 12 and 31 or 28, respectively). Since all belong to the Rio Negro catchment area, this suggests effective downstream water dispersal. Species composition and community dissimilarity are determined by local climate and land‐use patterns (Tables 2 and 3, Tables S4, S6–S9), and our results provide empirical evidence of underlying processes that shape the structure of metacommunities, such as environmental gradients (Neves et al., 2017; Oliviera‐Filho et al., 2015), dispersal limitation (MacArthur & Wilson, 1967), local endemism (Neves et al., 2017), and/or landscape structures, which we discuss below.

### 4.1 Native forests are largely disconnected

Even though they cover a small proportion of the country and are scattered, Uruguayan native forests harbor a high diversity of woody species (Figure 1a). The high species turnover, high local endemism, low frequency of species, and the increase in dissimilarity with geographic distance underline the low connectivity of these Uruguayan native forests (Table 1, Figure 3, Supporting information). Native forests of the Brazilian grasslands showed a similar high proportion of endemism compared to other forest types (Neves et al., 2017; Oliviera‐Filho et al., 2015).

Five out of six species with priority for conservation (i.e., *Actinostemon concolor*, *Butia odorata*, *Maytenus dasyclados*, *Phytolacca americana*, *Prosopis affinis*, *Xylosma schroederi*) were recorded only once, all were categorized with a zoochoric dispersal syndrome (Table S1). This indicates both the constrained distribution of some species and also local extinction of species that are nontolerant to disturbance (Zwiener et al., 2018). Other species were found only in regeneration layer. For example, *Casearia decandra*, which has priority conservation status, was registered twice in the regeneration layer. In addition to its medicinal value, *Casearia decandra* is recognized as a species with a high offering of resources to pollinators and birds (Narvaes et al., 2005). Although this species has been registered without recruitment problems in Brazil (Narvaes et al., 2005), there is evidence that germination of *Casearia decandra* is sensitive to drought (Rego et al., 2013). Our data indicate that *Casearia decandra* is currently recovering and recolonizing forests due to increasing precipitation in Uruguay. The woody understory *Actinostemon concolor* was registered once in the regeneration layer (Table S1). This species was classified as nontolerant to flooding and with high mortality in areas with high cover of herbs and litter (Bianchini et al., 2013). Population of *Maytenus dasyclados*, also recorded once in the regeneration layer, other studies showed that the species is decreasing in Southern Brazil as a result of anthropogenic fragmentation (Reichmann et al., 2017).

Similarly, the pattern observed in our study may result from a historically patchy distribution of disconnected native forests (Gautreau & Lezama, 2009), together with amplified disconnectivity due to ongoing land‐use change (e.g., Tiscornia et al., 2014) and/or historical processes of an expansion or reduction of native forest (e.g., Oliviera‐Filho et al., 2015). Community structure is shaped by processes that interact in spatio‐temporal scales such as dispersal processes, ecological drift, selection, and speciation (Vellend, 2016). In general, Uruguayan vegetation has been considered as a transitional zone between Pampas grasslands (Argentine) and the Chaco and Paranaense forests (Brazil) with an important tree species diversity (Haretche et al., 2012) and a high dissimilarity between native forest patches (e.g., Gautreau & Lezama, 2009). Even in a paleo‐ecological context, differences in community composition have been suggested within riparian forest (e.g., Mourelle et al., 2017).

### 4.2 Metacommunity assemblage follows longitude and precipitation pattern

The species distribution has previously been hypothesized to follow a latitudinal pattern, responding mainly to variation in temperature (Neves et al., 2017; Oliviera‐Filho et al., 2015). Our findings differ; communities were ordered in a longitudinal pattern, related predominately to variation of precipitation which influence the increasing of community dissimilarity from West to East (Table 2, Figure 3, Tables S6 and S7). Precipitation seasonality, precipitation of the driest quarter, and precipitation of the coldest quarter of the year showed stronger association with ordination of sites than climatic variables based on temperature (Table 2, Tables S4, S6 and S7). A similar response has been described for mixed forest at Southeast Brazil, representing a particularity within the Atlantic Forest (Marcilio‐Silva et al., 2017).

Historically, in the study area, grassland has dominated since Pleistocene, but since the Holocene, riparian forest has started to develop due to climate change, specifically the increase in precipitation regimes influenced by ENSO events in the past (Mourelle et al., 2017). Moisture is, therefore, an important factor that has permitted the development of woody species within an area dominated by grasslands (Mourelle et al., 2017). Studies conducted in Uruguay showed that regional environmental factors (Grela & Brussa, 2003; Lucas et al., 2017) and topographic factors (Gautreau & Lezama, 2009) shaped composition differences between different native forests. Woody species within a community should, therefore, be characterized based on adaptation to moisture, as hydrophilous, mesophilous, and subxerophilous species (Mourelle et al., 2017; Traversa‐Tejero & Alejano‐Monge, 2013).

Other studies have suggested that distribution ranges of tree species and forests type in the Pampean region has been influenced by the expansion and reduction of forests responding to ancient climate change (Mourelle et al., 2017). The current patchiness of native forest fragments (Oliviera‐Filho et al., 2015) has resulted in differences in composition with a high local endemism (Neves et al., 2017). We partially confirm this pattern, as we found marked boundaries of species distribution with species turnover across different native forests (Table 1, Table S3).

### 4.3 Forest types differ in community composition

Since the traditional classification of Uruguayan forests is based on topographic localization of forests within the landscape (Haretche et al., 2012), we expected to find correlations between species assemblages and geomorphological variables. However, neither elevation nor slope was linked to the structure of metacommunities nor community dissimilarity (Table 2, Tables S6 and S7).

Our research provides new insights into species composition of Uruguayan native forests. There is a clear distinction between riverine and hill forests with regard to metacommunity structures. We reveal a Clementsian pattern and a high species turnover within riverine forests at the regional scale (Table 1). In contrast, species turnover in hill forests is low and we observed a (quasi‐)nested stochastic species loss, which may be explained by historical processes (Mourelle et al., 2017; Oliviera‐Filho et al., 2015), species dispersal limitation (Neves et al., 2017), and/or by low species frequency (Leibold & Mikkelson, 2002) across the sites. Since the nested communities of hill forests are less distant from each other so better connected (Figure 1a; Table 1), they display higher similarity of species composition (see Figure 1a, Figure 2d–f and Table S10). 41 species were recorded only in riverine forests, 8 species occurred only in hill forests (e.g., *Calyptranthes concinna*, *Casearia sylvestris*, *Cephalanthus glabratus*, *Citronella paniculata*, *Ilex paraguariensis*, *Myrcia palustris*, *Myrsine parvula*, *Schinus engleri*; Supporting information), and 4 species only in park forests (e.g., *Bauhinia forticata*, *Butia yatay*, *Poncirus trifoliata*, *Prosopis affinis*; Table S1; see also Pozo & Säumel, 2018).

### 4.4 Zoochory as crucial agent

Phyto‐historical studies have postulated the forest expansion in the Holocene over grasslands after the last glacial maximum from hills and river sites as local forest refuges into the grasslands (Mourelle et al., 2017; Oliviera‐Filho et al., 2015). Beyond these phyto‐historical pattern (Lucas et al., 2017; Mourelle et al., 2017; Oliviera‐Filho et al., 2015; Zwiener et al., 2018), Uruguayan native forests are shaped by dispersal processes (e.g., Nores et al., 2005). Landscape changes can modify the richness and abundance of dispersal agents (e.g., Phifer et al., 2017), causing a decline in recruitment of species. In our study, 70 percent of the woody species were zoochorous species, which dominate local forest communities across Uruguay (Supporting information). Birds are long distance dispersers and can effectively connect species communities over long distances (Christianini & Oliveira, 2010). Zoochorous and riparian plant species cover markedly greater distances along the local riverine forests than anemochorous species and nonriparian species (Nores et al., 2005). In contrast to zoochory, hydrochory occurs mainly downstream. Although we expected a higher similarity along riverine forest and a higher degree of nestedness between forests, the similarity between riverine forests at the regional scale was low, suggesting an ecological filter that poses a barrier to dispersion: for example, the increase in productive areas (e.g., crop or timber plantations) limits the crossing or abundance of dispersing agents (e.g., birds). In general, crop or timber plantations are extensively managed with agrochemicals that are not friendly to the disperser (da Silva & Rossa‐Feres, 2017). In addition, Uruguay has lost between 35 and 45% of habitats with assigned priorities for biodiversity conservation of vertebrates and tree species (Brazeiro et al., 2020). Land‐use change generates environmental filters for species recruitment, directly impacting on source of propagules and dispersal vectors. This varies the composition between different native forest fragments.

### 4.5 Novel land‐use types alter metacommunity structures

Landscape heterogeneity in Uruguay increased through the emergence of novel land‐use patches in the originally dominant natural grasslands. Our results demonstrate the influence of a related disaggregation and enhanced shape‐complexity of land‐use patches on the woody communities’ composition (Table 2). In particular, timber plantations and crops strongly shape the metacommunity, species richness, and community dissimilarity of neighboring native forests (Table 3, Tables S8 and S9). The overall impact of the changed landscape pattern (i.e., all metrics; Tables S8 and S9) is the main driver of community dissimilarities compared to the environmental variables of our study (Table 2, Tables S4, S6 and S7).

Although, following regulation from government (MGAP, 2018), all areas covered by native forests are managed and conserved by private landowners, the increase in productive areas in the surroundings and the extinction probability of dispersal agents (e.g., Mortelliti & Lindenmayer, 2015) generates barriers to seed dispersal (e.g., Tomasevic & Estades, 2008). Landscape modification affects particular taxa and produces variation at the trophic levels (e.g., da Silva & Rossa‐Feres, 2017), including disappearance of dispersal agents (e.g., Mortelliti & Lindenmayer, 2015). There is evidence that, due to a lack of resources from the original habitats, landscape with afforestation increases nest predation of birds by generalists (e.g., Okada et al., 2019). This can reduce both abundance and richness of these dispersal agents (Phifer et al., 2017; Terborgh et al., 2008). Consequently, policies on biodiversity conservation need to include ecological dynamics of species interaction at landscape scale and extend their focus beyond nature reserves and take the presence new land‐use types (i.e., afforestation) into account.

### 4.6 Look over your neighbor's fence!

Our data highlight that pressure of neighborhood land uses was the predominant factor for species assemblages. The number of patch adjacency to native forest fragments (based on Interspersion and Juxtaposition Index) influenced the similarity between woody communities and the decline of species richness (Table 3). Other landscape metrics related to native forests (i.e., the number of native forest patches, mean Euclidean nearest neighbor distance of native forest patches, total area, perimeter‐area ratio, shape index) were not relevant for metacommunity structure, species richness, and community dissimilarity (Table 3, Tables S8 and S9).

Until now, even though the Uruguayan native forests experienced a long history of anthropogenic pressures, such as clear‐cutting to agriculture expansion and firewood (Brazeiro, 2018), few local studies have evaluated how native forests respond to adjacent land uses. We found an unbalance between the presence of adults and lacking juveniles, indicating limiting recruitments (Table S1). There is a high dissimilarity across woody communities due to diminishing of recruitment in different ways, and some evidences indicate that at local scale livestock reduce plant recruitment but not the species composition (Etchebarne & Brazeiro, 2016).

Current governmental measures of native forest protection focus on restrictions of logging and cutting within native forests, but do not address impacts of neighboring novel land uses. Our data underline the importance of redirecting the conservation paradigm from traditional reserve‐based approaches toward the landscape scale and integrating biodiversity targets in productive land uses (Donaldson et al., 2017). These could include the implementation of larger buffer zones from highly intensified land uses to native forests, the incorporation of native species in timber plantations, mixed species stands, mixed plantation buffer strips, and approaches to balance the coverage of young and older stands in order to reduce impacts of timber plantations (Pozo & Säumel, 2018).

The increase in productive land uses adjacent to native forest creates barriers to fauna that act as dispersal agents. This is of particular importance due to the predominance of zoochorous woody species in Uruguayan native forests. An increase in forest fragmentation is likely to result in a decline of frugivorous species, in negative effects on both animal and plant communities (e.g., Terborgh et al., 2008), and a lack of functional connectivity among native forest fragments (Ramos et al., 2020).

### 4.7 Conservation implications

Our findings suggest that strategies and planning for biodiversity conservation should consider synergies between at least two not mutually exclusive ways on landscape and species conservation. At landscape scale, focusing on conserving native forests and other supporting landscape structures that assure connectivity within and among native forest patches is crucial. At the local level on sites with rare target species, the creation of alliances between governmental institution and landowner plus incentives to biodiversity conservation provides opportunities to advance in species protection focused on those less tolerant to land‐use change. Biodiversity‐friendly farm planning will reduce pressure over nature near areas by buffer zones and connecting structures that do not need large areas.

At the landscape scale, since near patches share more species than distant patches, the most promising direction for species conservation is increasing connectivity to improve the movement of dispersal agent and to allow recruitment between native forests. As, at the regional scale, we found that Uruguayan native forests are highly diverse and dissimilar, land‐use planning at country level should recognize the great diversity of environment and its species. In particular, zones with a high risk of species extinction that could be harboring species with high conservation value should be identified. Finally, discussions about the contribution of historically patchy distribution of native forests to disconnectivity at landscape scale and to what extent land‐use change fosters disconnectivity, are likely to continue.

## CONFLICT OF INTEREST

The authors have no conflicts of interest to declare.

## AUTHOR CONTRIBUTIONS

**Leonardo R. Ramírez:** Data curation (equal); Formal analysis (equal); Investigation (equal); Methodology (equal); Validation (equal); Visualization (equal); Writing – original draft (equal); Writing – review & editing (equal). **Ina Säumel:** Conceptualization (lead); Data curation (equal); Formal analysis (equal); Funding acquisition (lead); Investigation (equal); Methodology (equal); Project administration (equal); Resources (lead); Supervision (equal); Validation (equal); Writing – original draft (equal); Writing – review & editing (equal).

## Supporting information

Supplementary Material

## Data Availability

The authors confirm that the data supporting the findings of this study are available within the article and in its supplementary materials. Further data are available from the corresponding author, [IS], upon reasonable request.
