# Supplementary material for: Native forest metacommunity structures in Uruguay shaped by novel land‐use types in their surroundings
Source: Ecol Evol. 2022 Mar 6;12(3):e8700. doi: 10.1002/ece3.8700 (PMC8928906; doi:10.1002/ece3.8700)
Supplement: Supplementary file 1 — Supplementary Material [file ECE3-12-e8700-s001.docx]

**Supporting information**

**Table S1.** Frequency and traits of woody species recorded at 32 permanent plots across Uruguay. AF: absolute frequency, RF: relative frequency (%) and CRF: cumulative relative frequency. Species ordered according to absolute frequency (AF) of Total from higher to lowest values. Presence based on forest type: R: riverine, H: Hill and P: park. Dispersal Syndrome (zoochory, anemochory, autochory), conservation priority and origin are given (n/a: non-information available). * shrubs, ^+^ mistletoe and ° liana (see material and methods).

| Family | Specie | Total | | | Adults | | | Juveniles | | | Presence based on forest type | Dyspersal  syndrome | Conservation  priority | Origin |
| --- | --- | --- | --- | --- | --- | --- | --- | --- | --- | --- | --- | --- | --- | --- |
|  |  | AF | RF | CRF | AF | RF | CRF | AF | RF | CRF |  |  |  |  |
| Sapindaceae | *Allophylus edulis* | 31 | 97 | 6 | 28 | 88 | 8.7 | 30 | 94 | 6.8 | RHP | zoochory | non-priority | native |
| Rhamnaceae | *Scutia buxifolia* | 30 | 94 | 6 | 26 | 81 | 8.0 | 22 | 69 | 5.0 | RHP | zoochory | non-priority | native |
| Myrtaceae | *Blepharocalyx salicifolius* | 29 | 91 | 6 | 20 | 63 | 6.2 | 28 | 88 | 6.4 | RHP | zoochory | non-priority | native |
| Celastraceae | *Maytenus ilicifolia* | 21 | 66 | 4 | 1 | 3 | 0.3 | 21 | 66 | 4.8 | RH | zoochory | non-priority | native |
| Cannabaceae | *Celtis tala* | 19 | 59 | 4 | 10 | 31 | 3.1 | 18 | 56 | 4.1 | RHP | zoochory | non-priority | native |
| Euphorbiaceae | *Sebastiania brasiliensis* | 18 | 56 | 3 | 16 | 50 | 5.0 | 15 | 47 | 3.4 | RH | autochory | non-priority | native |
| Myrtaceae | *Myrcianthes cisplatensis* | 18 | 56 | 3 | 16 | 50 | 5.0 | 11 | 34 | 2.5 | RH | zoochory | non-priority | native |
| Thymelaeaceae | *Daphnopsis racemosa** | 18 | 56 | 3 | 1 | 3 | 0.3 | 18 | 56 | 4.1 | RH | zoochory | non-priority | native |
| Euphorbiaceae | *Sebastiania commersoniana* | 17 | 53 | 3 | 16 | 50 | 5.0 | 16 | 50 | 3.7 | RH | autochory | non-priority | native |
| Sapotaceae | *Pouteria salicifolia* | 17 | 53 | 3 | 15 | 47 | 4.6 | 13 | 41 | 3.0 | RH | zoochory | non-priority | native |
| Myrtaceae | *Eugenia uniflora* | 16 | 50 | 3 | 12 | 38 | 3.7 | 16 | 50 | 3.7 | RHP | zoochory | non-priority | native |
| Myrtaceae | *Myrrhinium atropurpureum* | 14 | 44 | 3 | 8 | 25 | 2.5 | 8 | 25 | 1.8 | RH | zoochory | non-priority | native |
| Anacardiaceae | *Lithraea brasiliensis* | 12 | 38 | 2 | 11 | 34 | 3.4 | 8 | 25 | 1.8 | RH | zoochory | non-priority | native |
| Primulaceae | *Myrsine laetevirens* | 11 | 34 | 2 | 9 | 28 | 2.8 | 10 | 31 | 2.3 | RH | zoochory | non-priority | native |
| Anacardiaceae | *Schinus longifolia* | 11 | 34 | 2 | 8 | 25 | 2.5 | 6 | 19 | 1.4 | RHP | zoochory | non-priority | native |
| Arecaceae | *Syagrus romanzoffiana* | 11 | 34 | 2 | 3 | 9 | 0.9 | 10 | 31 | 2.3 | RH | zoochory | non-priority | native |
| Myrtaceae | *Eugenia uruguayensis* | 10 | 31 | 2 | 10 | 31 | 3.1 | 9 | 28 | 2.1 | RH | zoochory | non-priority | native |
| Verbenaceae | *Citharexylum montevidense* | 9 | 28 | 2 | 5 | 16 | 1.5 | 9 | 28 | 2.1 | RH | zoochory | non-priority | native |
| Cannabaceae | *Celtis iguanaea** | 8 | 25 | 2 |  |  |  | 8 | 25 | 1.8 | RHP | zoochory | non-priority | native |
| Smilacaceae | *Smilax campestris°* | 8 | 25 | 2 |  |  |  | 8 | 25 | 1.8 | RH | n/a | n/a | native |
| Fabaceae | *Calliandra tweedii** | 7 | 22 | 1 | 1 | 3 | 0.3 | 7 | 22 | 1.6 | RH | zoochory | n/a | native |
| Lythraceae | *Heimia salicifolia** | 7 | 22 | 1 |  |  |  | 7 | 22 | 1.6 | RHP | n/a | non-priority | native |
| Fabaceae | *Erythrina crista-galli* | 6 | 19 | 1 | 6 | 19 | 1.9 | 3 | 9 | 0.7 | RH | autochory | non-priority | native |
| Loranthaceae | *Tripodanthus acutifolius^+^* | 6 | 19 | 1 | 6 | 19 | 1.9 |  |  |  | RH | zoochory | non-priority | native |
| Lauraceae | *Ocotea acutifolia* | 6 | 19 | 1 | 4 | 13 | 1.2 | 5 | 16 | 1.1 | RH | zoochory | non-priority | native |
| Oleaceae | *Ligustrum lucidum* | 6 | 19 | 1 | 4 | 13 | 1.2 | 4 | 13 | 0.9 | RHP | zoochory | non-evaluated | Exot |
| Myrtaceae | *Myrcianthes pungens* | 6 | 19 | 1 | 4 | 13 | 1.2 | 4 | 13 | 0.9 | RH | zoochory | non-priority | native |
| Berberidaceae | *Berberis laurina** | 6 | 19 | 1 | 2 | 6 | 0.6 | 5 | 16 | 1.1 | RH | zoochory | non-priority | native |
| Rubiaceae | *Guettarda uruguensis** | 5 | 16 | 1 | 4 | 13 | 1.2 | 5 | 16 | 1.1 | R | zoochory | non-priority | native |
| Salicaceae | *Salix humboldtiana* | 5 | 16 | 1 | 4 | 13 | 1.2 | 3 | 9 | 0.7 | RH | anemochory | non-priority | native |
| Sapindaceae | *Cupania vernalis* | 5 | 16 | 1 | 3 | 9 | 0.9 | 5 | 16 | 1.1 | RH | zoochory | non-priority | native |
| Salicaceae | *Xylosma tweediana* | 5 | 16 | 1 | 2 | 6 | 0.6 | 4 | 13 | 0.9 | R | zoochory | non-priority | native |
| Fabaceae | *Gleditsia triacanthos* | 4 | 13 | 1 | 3 | 9 | 0.9 | 4 | 13 | 0.9 | RP | zoochory | non-evaluated | Exot |
| Lamiaceae | *Vitex megapotamica* | 4 | 13 | 1 | 3 | 9 | 0.9 | 4 | 13 | 0.9 | RH | zoochory | non-priority | native |
| Malvaceae | *Luehea divaricata* | 4 | 13 | 1 | 3 | 9 | 0.9 | 3 | 9 | 0.7 | RH | anemochory | non-priority | native |
| Lauraceae | *Nectandra megapotamica* | 4 | 13 | 1 | 2 | 6 | 0.6 | 4 | 13 | 0.9 | RH | zoochory | non-priority | native |
| Verbenaceae | *Aloysia gratissima** | 4 | 13 | 1 | 1 | 3 | 0.3 | 4 | 13 | 0.9 | R | autochory | non-priority | native |
| Fabaceae | *Acacia caven* | 3 | 9 | 1 | 3 | 9 | 0.9 | 2 | 6 | 0.5 | R | zoochory | non-priority | native |
| Fabaceae | *Vachellia caven** | 3 | 9 | 1 | 3 | 9 | 0.9 | 2 | 6 | 0.5 | R | zoochory | n/a | native |
| Quillajaceae | *Quillaja brasiliensis* | 3 | 9 | 1 | 2 | 6 | 0.6 | 3 | 9 | 0.7 | R | anemochory | non-priority | native |
| Polygonaceae | *Ruprechtia salicifolia* | 3 | 9 | 1 | 2 | 6 | 0.6 | 3 | 9 | 0.7 | R | n/a | non-priority | native |
| Oleaceae | *Ligustrum sinense* | 3 | 9 | 1 | 2 | 6 | 0.6 | 3 | 9 | 0.7 | R | zoochory | n/a | Exot |
| Sapindaceae | *Matayba elaeagnoides* | 3 | 9 | 1 | 2 | 6 | 0.6 | 2 | 6 | 0.5 | RH | zoochory | non-priority | native |
| Lauraceae | *Ocotea puberula* | 3 | 9 | 1 | 2 | 6 | 0.6 | 2 | 6 | 0.5 | RH | zoochory | non-priority | native |
| Styracaceae | *Styrax leprosus* | 3 | 9 | 1 | 2 | 6 | 0.6 | 2 | 6 | 0.5 | RH | zoochory | non-priority | native |
| Primulaceae | *Myrsine coriacea* | 3 | 9 | 1 | 1 | 3 | 0.3 | 3 | 9 | 0.7 | RH | zoochory | non-priority | native |
| Polygonaceae | *Ruprechtia laxiflora** | 3 | 9 | 1 | 1 | 3 | 0.3 | 3 | 9 | 0.7 | R | anemochory | non-priority | native |
| Rosaceae | *Prunus subcoriacea* | 3 | 9 | 1 | 1 | 3 | 0.3 | 2 | 6 | 0.5 | RH | zoochory | non-priority | native |
| Fabaceae | *Acacia bonariensis* | 3 | 9 | 1 |  |  |  | 3 | 9 | 0.7 | R | anemochory | non-priority | native |
| Rutaceae | *Zanthoxylum rhoifolium* | 3 | 9 | 1 |  |  |  | 3 | 9 | 0.7 | RH | zoochory | non-priority | native |
| Primulaceae | *Myrsine parvula* | 2 | 6 | 0.4 | 2 | 6 | 0.6 | 2 | 6 | 0.5 | H | zoochory | non-priority | native |
| Fabaceae | *Parapiptadenia rigida* | 2 | 6 | 0.4 | 2 | 6 | 0.6 | 2 | 6 | 0.5 | RP | anemochory | non-priority | native |
| Anacardiaceae | *Schinus molle* | 2 | 6 | 0.4 | 2 | 6 | 0.6 | 2 | 6 | 0.5 | RP | zoochory | non-priority | native |
| Anacardiaceae | *Lithraea molleoides* | 2 | 6 | 0.4 | 2 | 6 | 0.6 |  |  |  | RH | zoochory | n/a | native |
| Anacardiaceae | *Schinus lentiscifolius* | 2 | 6 | 0.4 | 2 | 6 | 0.6 |  |  |  | R | zoochory | non-priority | native |
| Salicaceae | *Azara uruguayensis* | 2 | 6 | 0.4 | 1 | 3 | 0.3 | 2 | 6 | 0.5 | R | n/a | non-priority | native |
| Rhamnaceae | *Colletia paradoxa** | 2 | 6 | 0.4 | 1 | 3 | 0.3 | 2 | 6 | 0.5 | RH | autochory | non-priority | native |
| Santalaceae | *Jodina rhombifolia* | 2 | 6 | 0.4 | 1 | 3 | 0.3 | 2 | 6 | 0.5 | RH | zoochory | non-priority | native |
| Primulaceae | *Myrsine venosa* | 2 | 6 | 0.4 | 1 | 3 | 0.3 | 2 | 6 | 0.5 | RH | zoochory | non-priority | native |
| Aquifoliaceae | *Ilex paraguariensis* | 2 | 6 | 0.4 | 1 | 3 | 0.3 | 1 | 3 | 0.2 | H | zoochory | priority | native |
| Myrtaceae | *Myrceugenia glaucescens* | 2 | 6 | 0.4 | 1 | 3 | 0.3 | 1 | 3 | 0.2 | RP | zoochory | non-priority | native |
| Salicaceae | *Casearia decandra* | 2 | 6 | 0.4 |  |  |  | 2 | 6 | 0.5 | RH | zoochory | priority | native |
| Rubiaceae | *Psychotria carthagenensis** | 2 | 6 | 0.4 |  |  |  | 2 | 6 | 0.5 | R | zoochory | non-priority | native |
| Fabaceae | *Senna corymbosa* | 2 | 6 | 0.4 |  |  |  | 2 | 6 | 0.5 | R | n/a | non-priority | native |
| Symplocaceae | *Symplocos uniflora* | 2 | 6 | 0.4 |  |  |  | 2 | 6 | 0.5 | RH | zoochory | non-priority | native |
| Myrtaceae | *Calyptranthes concinna* | 1 | 3 | 0.2 | 1 | 3 | 0.3 | 1 | 3 | 0.2 | H | zoochory | non-priority | native |
| Salicaceae | *Casearia sylvestris* | 1 | 3 | 0.2 | 1 | 3 | 0.3 | 1 | 3 | 0.2 | H | zoochory | non-priority | native |
| Rubiaceae | *Cephalanthus glabratus** | 1 | 3 | 0.2 | 1 | 3 | 0.3 | 1 | 3 | 0.2 | H | n/a | non-priority | native |
| Boraginaceae | *Cordia americana* | 1 | 3 | 0.2 | 1 | 3 | 0.3 | 1 | 3 | 0.2 | R | anemochory | n/a | native |
| Asteraceae | *Gochnatia polymorpha* | 1 | 3 | 0.2 | 1 | 3 | 0.3 | 1 | 3 | 0.2 | R | anemochory | non-priority | native |
| Moraceae | *Morus alba* | 1 | 3 | 0.2 | 1 | 3 | 0.3 | 1 | 3 | 0.2 | R | n/a | n/a | Exot |
| Myrtaceae | *Myrcia palustris* | 1 | 3 | 0.2 | 1 | 3 | 0.3 | 1 | 3 | 0.2 | H | zoochory | n/a | native |
| Phyllanthaceae | *Phyllanthus sellowianus** | 1 | 3 | 0.2 | 1 | 3 | 0.3 | 1 | 3 | 0.2 | R | zoochory | non-priority | native |
| Fabaceae | *Prosopis affinis* | 1 | 3 | 0.2 | 1 | 3 | 0.3 | 1 | 3 | 0.2 | P | zoochory | priority | native |
| Myrtaceae | *Acca sellowiana* | 1 | 3 | 0.2 | 1 | 3 | 0.3 |  |  |  | R | zoochory | non-priority | native |
| Fabaceae | *Bauhinia forficata* | 1 | 3 | 0.2 | 1 | 3 | 0.3 |  |  |  | P | autochory | non-priority | native |
| Arecaceae | *Butia odorata* | 1 | 3 | 0.2 | 1 | 3 | 0.3 |  |  |  | R | zoochory | priority | native |
| Cardiopteridaceae | *Citronella gongonha* | 1 | 3 | 0.2 | 1 | 3 | 0.3 |  |  |  | R | zoochory | non-priority | native |
| Escalloniaceae | *Escallonia bifida* | 1 | 3 | 0.2 | 1 | 3 | 0.3 |  |  |  | R | autochory | non-priority | native |
| Bignoniaceae | *Handroanthus impetiginosus* | 1 | 3 | 0.2 | 1 | 3 | 0.3 |  |  |  | R | anemochory | n/a | native |
| Phytolaccaceae | *Phytolacca dioica* | 1 | 3 | 0.2 | 1 | 3 | 0.3 |  |  |  | R | zoochory | non-priority | native |
| Euphorbiaceae | *Sapium haematospermum* | 1 | 3 | 0.2 | 1 | 3 | 0.3 |  |  |  | R | zoochory | non-priority | native |
| Anacardiaceae | *Schinus engleri** | 1 | 3 | 0.2 | 1 | 3 | 0.3 |  |  |  | H | n/a | non-priority | native |
| Rutaceae | *Zanthoxylum fagara* | 1 | 3 | 0.2 | 1 | 3 | 0.3 |  |  |  | R | zoochory | non-priority | native |
| Euphorbiaceae | *Actinostemon concolor* | 1 | 3 | 0.2 |  |  |  | 1 | 3 | 0.2 | R | autochory | priority | native |
| Arecaceae | *Butia yatay* | 1 | 3 | 0.2 |  |  |  | 1 | 3 | 0.2 | P | zoochory | non-priority | native |
| Cannabaceae | *Celtis ehrenbergiana** | 1 | 3 | 0.2 |  |  |  | 1 | 3 | 0.2 | R | zoochory | n/a | native |
| Solanaceae | *Cestrum parqui** | 1 | 3 | 0.2 |  |  |  | 1 | 3 | 0.2 | R | zoochory | non-priority | native |
| Sapotaceae | *Chrysophyllum gonocarpum* | 1 | 3 | 0.2 |  |  |  | 1 | 3 | 0.2 | R | zoochory | non-priority | native |
| Cardiopteridaceae | *Citronella paniculata* | 1 | 3 | 0.2 |  |  |  | 1 | 3 | 0.2 | H | zoochory | non-priority | native |
| Rhamnaceae | *Discaria americana** | 1 | 3 | 0.2 |  |  |  | 1 | 3 | 0.2 | R | autochory | non-priority | native |
| Celastraceae | *Maytenus dasyclados* | 1 | 3 | 0.2 |  |  |  | 1 | 3 | 0.2 | R | zoochory | priority | native |
| Meliaceae | *Melia azedarach* | 1 | 3 | 0.2 |  |  |  | 1 | 3 | 0.2 | R | zoochory | n/a | Exot |
| Phytolaccaceae | *Phytolacca americana** | 1 | 3 | 0.2 |  |  |  | 1 | 3 | 0.2 | R | zoochory | priority | native |
| Rutaceae | *Poncirus trifoliata* | 1 | 3 | 0.2 |  |  |  | 1 | 3 | 0.2 | P | n/a | non-priority | Exot |
| Myrtaceae | *Psidium luridum** | 1 | 3 | 0.2 |  |  |  | 1 | 3 | 0.2 | R | n/a | n/a | native |
| Myrtaceae | *Psidium salutare** | 1 | 3 | 0.2 |  |  |  | 1 | 3 | 0.2 | R | n/a | n/a | native |
| Rosaceae | *Pyracantha coccinea** | 1 | 3 | 0.2 |  |  |  | 1 | 3 | 0.2 | R | zoochory | non-evaluated | Exot |
| Solanaceae | *Solanum mauritianum* | 1 | 3 | 0.2 |  |  |  | 1 | 3 | 0.2 | R | zoochory | non-priority | native |
| Solanaceae | *Vassobia breviflora* | 1 | 3 | 0.2 |  |  |  | 1 | 3 | 0.2 | R | zoochory | non-priority | native |
| Salicaceae | *Xylosma schroederi* | 1 | 3 | 0.2 |  |  |  | 1 | 3 | 0.2 | R | zoochory | priority | native |

**Reference – Table S2**

1. **Dispersal syndrome**

Barberis, I. M., Batista, W. B., Pire, E. F., Lewis, J. P. & León, R. J. (2002). Woody population distribution and environmental heterogeneity in a Chaco forest, Argentina. *Journal of Vegetation Science*, 13, 607-614.

Bianchini, E., Araújo, C.G.D., Green, M. & Pimenta, J.A. (2013). Demography and Structures Population of *Actinostemon concolor* (Spreng.) Müll. Arg. Euphorbiaceae) in flooded areas in southern Brazil. *Brazilian Archives of Biology and Technology*, 56(1), 69-79.

Bravo, S.P. & Sallenave, A. (2003). Foraging behaviour and activity patterns of *Alouatta caraya* in the northeastern Argentinean flooded forest. *International Journal of Primatology*, 24, 825-846.

Budke, J.C., Athayde, E.A., Giehl, E.L.H., Zachia, R.A. & Eisenger, S.M. (2005). Composição florística e estratégias de dispersão de espécies lenhosasem uma floresta ribeirinha, arroio Passo das Tropas, Santa Maria, RS,Brasil. *Iheringia*, 60(1), 17-24.

Cabral, A. C., De Miguel, J. M., Rescia, A. J., Schmitz, M. F., & Pineda, F. D. (2003). Shrub encroachment in Argentinean savannas. *Journal of Vegetation Science*, 14(2), 145-152.

da Silva, A., Higuchi, P., Guerra Sobral, M., Negrini, M., Buzzi Júnior, F., Bento, … Dalla Rosa, A. (2017). Organização da comunidade e estrutura filogenética do componente arbóreo de um fragmento de floresta nebular no planalto catarinense. *Ciência Florestal*, 27 (1), 129-141.

D'Ambrogio, A., & Medan, D. (1993). Comportamiento reproductivo de *Colletia paradoxa* (Rhamnaceae). *Darwiniana*, 32(1-4), 1-14.

de la Peña, M.R. & Pensiero, J.F. (2003). Contribución de la flora en los hábitos alimentarios de las aves en un bosque del centro de la provincia de Santa Fe, Argentina. *Ornitología Neotropical*, 14, 499-513.

de Noir, F.A., Bravo, S., Abdala, R. (2002). Mecanismos de dispersión de algunas especies de leñosas nativas del Chaco Occidental y Serrano. *Quebracho*, 9,140-150.

Debussche, M. & Isenmann, P. (1994). Bird-dispersed seed rain and seedling establishment in patchy Mediterranean vegetation. *Oikos*, 69, 414–426.

Dennis, A.J., Schupp, E.W., Green, R.J., & Westcott, D.A. (2007). *Seed dispersal: theory and its application in a changing world*. CAB International Press, Wallingford, UK, 684 pp.

Díaz Vélez, M.C., Ferreras, A.E., Silva W.R. & Galetto, L. (2017). Does avian gut passage favour seed germination of woody species of the Chaco Serrano Woodland in Argentina? *Botany*, 95(5), 493-501.

Diaz-Martin, Z., Swamy, V., Terborgh, J., Alvarez-Loayza, P. & Cornejo, F. (2014). Identifying keystone plant resources in an Amazonian forest using a long-term fruit-fall record. *Journal of Tropical Ecology*, 30, 291-301.

Donaldson, L.; Wilson, R.J.; Maclean, I.M.D. Old concepts, new challenges: adapting landscape-scale conservation to the twenty-first century. *Biodiversity and Conservation*. 2017, 26, 527–552.

Gaiero, P., Mazzella, C., Agostini, G., Bertolazzi, M & Rossato, M. (2011). Genetic diversity among endangered Uruguayan populations of *Butia* Becc. species based on ISSR. *Plant Systematics and Evolution*, 292(1-2), 105-116.

Galetti, M. & Pedroni, F. (1994). Seasonal diet of capuchin monkeys (*Cebus apella*) in a semideciduous forest in south-east Brazil. *Journal of Tropical Ecology*, 10(1), 27-39.

Gauer, L. & Cavalli‐Molina, S. (2000). Genetic variation in natural populations of maté (*Ilex paraguariensis* A. St.‐Hil., Aquifoliaceae) using RAPD markers. *Heredity*, 84, 647-656.

Howard, L.F. & Minnich, R.A. (1989). The introduction and naturalization of *Schinus molle* (pepper tree) in Riverside, California. *Landscape and Urban Planning*, 18(2), 77-95.

Kattge, J., Diaz, S., Lavorel, S., Prentice, I. C., Leadley, P., Bönisch, … & Wirth, C. (2011). TRY - a global database of plant traits. *Global Change Biology*, 17, 2905-2935.

Kuhlmann, M.P. (2016). *Estratégias de dispersão de sementes no Bioma Cerrado: considerações ecológicas e filogenéticas*. Tese Doutorado em Botanica, Universidade de Brasília, available in <https://core.ac.uk/download/pdf/80745603.pdf>.

Martins, J. R., Edvaldo, A. A. S., Alvarenga, A. A., Rodrigues, A. C., Ribeiro, D. E., & Toorop, P. E. (2015). Seedling survival of *Handroanthus impetiginosus* (Mart ex DC) Mattos in a semi-arid environment through modified germination speed and post-germination desiccation tolerance. *Brazilian Journal of Biology*, 75(4), 812-820.

Martins, V.F., Cazotto, L.P.D. & dos Santos, F.A.M. (2014). Dispersal spectrum of four forest types along an altitudinal range of Brazilian Atlantic Rain Forest. *Biota Neotropica*, 14, 1-22.

McDonnell, M.J., Stiles, E.W., Cheplick, G.P. & Armesto J.J. (1984). Bird-dispersal of *Phytolacca americana* L. and the influence of fruit removal on subsequent fruit development. *American Journal of Botany*, 71, 895– 901.

Medan, D. (1993). Breeding system and maternal success of a perennial hermaphrodite, *Discaria americana* (Rhamnaceae). *New Zealand Journal of Botany*, 31, 175-184.

Reid, S. & Armesto, J.J. (2011). Avian gut-passage effects on seed germination of shrubland species in Mediterranean central Chile. *Journal of Plant Ecology*, 212, 1-10.

Saravy, F.P., de Freitas, P.J., Lage, M.A., Leite, S.J., Braga, L.F. & Sousa, M.P. (2003). Síndrome de dispersão em estratos arbóreos em um fragmento de floresta ombrófila aberta e densa em Alta Floresta - MT. *Alta Floresta*, 2(1), 1-12.

Schor, J., Farwig, N., & Berens, D. G. (2015). Intensive land-use and high native fruit availability reduce fruit removal of the invasive *Solanum mauritianum* in South Africa. *South African Journal of Botany*, 96, 6-12.

Scipioni, M.C., Galvão, F. & Longhi, S.J. (2013). Composição florística e estratégias de dispersão e regeneração de grupos florísticos em florestas estacionais deciduais no Rio Grande do Sul. Floresta, *Curitiba*, 43(2), 241-254.

Streit, H., Carlucci, M.B., Bergamin, R.S., Pillar, V.D. & Duarte, L.D.S. (2014). Patterns of diaspore functional diversity in Araucaria Forest successional stages in extreme southern Brazil. Revista Brasileira de Biociências, 12(2), 106-114.

Toniato, M.T.Z. & de Oliveira-Filho A.T. (2004). Variations in tree community composition and structure in a fragment of tropical semi deciduous forest in south eastern Brazil related to different human disturbance histories. *Forest Ecology and Management*, 198, 319-339.

1. **Conservation priority**

MVOTMA-MGAP-SNAP. (2011). Base de datos de especies. Proyecto fortalecimiento del proceso de implementación del sistema nacional de áreas protegidas del Uruguay (URU/06/G34). Available in https://www.dinama.gub.uy/especies/

Soutullo, A., Clavijo, C. & Martínez-Lanfranco, J.A. (2013). *Especies prioritarias para la conservación en Uruguay. Vertebrados, moluscos continentales y plantas vasculares*. SNAP/DINAMA/MVOTMA y DICYT/ MEC, Montevideo. 222 pp.

1. **Origin**

Laboratório de Fitoecologia e Fitogeografia - Universidade Federal do Rio Grande do Sul. (2010). Projeto Flora digital do Rio Grande do Sul e de Santa Catarina. (Giehl E.L.H. coord.). Available in http://www.ufrgs.br/fitoecologia/florars/index.php?pag=contato.php.

MVOTMA-MGAP-SNAP. (2011). Base de datos de especies. *Proyecto fortalecimiento del proceso de implementación del sistema nacional de áreas protegidas del Uruguay* (URU/06/G34). Available in https://www.dinama.gub.uy/especies/.

**Table S2.** Variables used in the analysis. A: Elements of metacommunity structure (based on Leibold & Mikkelson 2002 and Presley et al., 2010). B: Composition (Jaccard Index, based on Jaccard 1912^[[1]](#footnote-1)^) and diversity. C: Landscape metrics (landscape, class and patch level, based on McGarigal et al. 2012). D: Climate and geographic data (based on Fick & Hijmans 2017 and MVOTMA 2017).

| Variables | | Description |
| --- | --- | --- |
| A | Coherence | Number of interruptions in species distribution across the sites. When coherence is negative or not significant, the metacommunity follows a checkboard or random pattern respectively. When coherence is statistically significant and positive (p<0.05; less embedded absences than expected by chance), the metacommunity is classified into six basic structures evaluating turnover and boundary clumping. |
|  | Species turnover | Number of species replacements between two sites. When turnover is statistically significant and negative (p<0.05; less replacements than expected by chance), the metacommunity can follow some nested pattern (i.e. evenly-spaced, clumped or random). When turnover is statistically significant and positive (p<0.05; with more replacements than expected by chance), the metacommunity is classified as a Clementsian, Gleasonian or evenly-spaced pattern. |
|  | Morisita overlap index | Boundaries in species composition across two or more sites based on Morisita overlap index. The Morisita index (MI) needs to be evaluated to determine boundary clumping between different woody communities (if MI > 1, a Clementsian structure and if MI < 1, an evenly spaced structure) |
| B | Jaccard Index (J) | Composition (di)similarity between sites |
|  | Species Richness | Number of species per site |
| C | Landscape | Number of patches (total number of patches in the landscape without considering the identity of the land use type); Landscape shape index (standardized measure of total edge that adjusts for the size of the landscape, where index increases without limit as landscape shape becomes more irregular and/or as the length of edge within the landscape increases); Shannon's evenness index (distribution of area among patch types where larger values mean higher landscape diversity); Aggregation Index (frequency with which different pairs of patch types appear side-by-side on the map). |
|  | Class | Percentage of the landscape occupied by each land use type; Number of native forest patches within the landscape; Interspersion and juxtaposition index (measure at class level based on patch adjacencies); Euclidean nearest neighbor distance (mean of the shortest straight-line distance between all native forest patches by landscape). |
|  | Patch | Total area of the patch where the plot is located; Perimeter area ratio of this patch as a measure of shape complexity; Shape index of these patch as a measure of compactness |
| D | Climate & Geographic | Annual Mean Temperature; Temperature Seasonality (standard deviation *100); Mean Temperature of Wettest Quarter; Mean Temperature of Driest Quarter; Mean Temperature of Warmest Quarter; Mean Temperature of Coldest Quarter; Annual Precipitation; Precipitation Seasonality (Coefficient of Variation); Precipitation of Wettest Quarter; Precipitation of Driest Quarter; Precipitation of Warmest Quarter; Precipitation of Coldest Quarter; Elevation (meter above sea level); slope (%); Longitude (UTM); Latitude (UTM); Distance between sites (d) |

**Table S3.** Matrix ordination by reciprocal averaging of both age classes together (juveniles and adults) for 32 native fragments under study (rows) and species recorded (columns). Black cell = presence and White cells = absence.

**Table S4.** Pearson’s correlation between the metacommunity structure based on first axis of ordination (reciprocal averaging) and species diversity of woody species arrangement and richness (all species, adult and juvenile individuals) versus longitude/latitude, meter above sea level, slope and bioclimatic variables. p-value in bold indicate significance with *p* < 0.05.

| Variable | First Axis Ordination | | | | | | Species Richness | | | | | |
| --- | --- | --- | --- | --- | --- | --- | --- | --- | --- | --- | --- | --- |
|  | All woody species | | Adults | | Juveniles | | All woody species | | Adults | | Juveniles | |
|  | Pearson | *p* | Pearson | *p* | Pearson | *p* | Pearson | *p* | Pearson | *p* | Pearson | *p* |
| Longitude (UTM) | **0.59** | **0.0004** | **0.56** | **0.0009** | **0.70** | **<0.0001** | **0.67** | **<0.0001** | **0.79** | **<0.0001** | **0.51** | **0.0031** |
| Latitude (UTM) | -0.22 | 0.2278 | -0.15 | 0.4051 | -0.07 | 0.7054 | 0.23 | 0.2075 | 0.07 | 0.6872 | 0.27 | 0.1361 |
| Meter above sea level | 0.04 | 0.8135 | 0.14 | 0.4387 | 0.11 | 0.5508 | 0.04 | 0.8135 | 0.14 | 0.4387 | 0.11 | 0.5508 |
| Slope (%) | 0.13 | 0.4884 | 0.08 | 0.6704 | 0.11 | 0.5332 | 0.13 | 0.4884 | 0.08 | 0.6704 | 0.11 | 0.5332 |
| Annual Mean Temperature | -0.30 | 0.1004 | -0.26 | 0.1542 | -0.17 | 0.3553 | 0.12 | 0.5214 | -0.10 | 0.5742 | 0.23 | 0.2006 |
| Temperature Seasonality | **-0.40** | **0.0252** | **-0.36** | **0.0437** | **-0.45** | **0.0091** | **-0.53** | **0.0017** | **-0.66** | **<0.0001** | **-0.36** | **0.0406** |
| Mean Temperature of Wettest Quarter | **-0.47** | **0.0067** | **-0.42** | **0.0174** | **-0.47** | **0.0063** | **-0.53** | **0.0020** | **-0.59** | **0.0004** | **-0.39** | **0.0295** |
| Mean Temperature of Driest Quarter | **0.50** | **0.0033** | **0.45** | **0.0105** | **0.54** | **0.0015** | **0.57** | **0.0007** | **0.68** | **<0.0001** | **0.44** | **0.0119** |
| Mean Temperature of Warmest Quarter | **-0.36** | **0.0412** | -0.32 | 0.0722 | -0.29 | 0.1116 | -0.09 | 0.6379 | -0.30 | 0.0930 | 0.06 | 0.7314 |
| Mean Temperature of Coldest Quarter | -0.14 | 0,4515 | -0.11 | 0.5596 | 0.04 | 0.8111 | **0.36** | **0.0404** | 0.20 | 0.2841 | **0.42** | **0.0176** |
| Annual Precipitation | 0.06 | 0,7339 | 0.16 | 0.3789 | 0.28 | 0.1250 | 0.34 | 0.0576 | **0.37** | **0.0382** | 0.22 | 0.2158 |
| Precipitation Seasonality | **-0.59** | **0.0004** | **-0.59** | **0.0004** | **-0.66** | **<0.0001** | -0.27 | 0.1424 | **-0.52** | **0.0021** | -0.12 | 0.5146 |
| Precipitation of Wettest Quarter | **-0.44** | **0.0115** | -0.33 | 0.0633 | -0.34 | 0.0564 | -0.16 | 0.3798 | -0.28 | 0.1155 | -0.12 | 0.5258 |
| Precipitation of Driest Quarter | **0.56** | **0.0008** | **0.58** | **0.0005** | **0.70** | **<0.0001** | **0.41** | **0.0212** | **0.62** | **0.0002** | 0.25 | 0.1630 |
| Precipitation of Warmest Quarter | -0.32 | 0,0712 | -0.21 | 0.2435 | -0.15 | 0.4242 | 0.001 | 0.9940 | -0.11 | 0.5495 | 0.05 | 0.7905 |
| Precipitation of Coldest Quarter | **0.63** | **0.0001** | **0.61** | **0.0002** | **0.73** | **<0.0001** | **0.61** | **0.0002** | **0.76** | **<0.0001** | **0.43** | **0.0129** |

**Table S5.** Department and site each permanent plots (ID) across Uruguay and environmental data (topographic and bioclimatic variables). ID = code native forest fragments (Fig. 1). Elevation = meter above sea level. bio1 = Annual Mean Temperature. bio4 = Temperature Seasonality (standard deviation *100). bio8 = Mean Temperature of Wettest Quarter. bio9 = Mean Temperature of Driest Quarter. bio10 = Mean Temperature of Warmest Quarter. bio11 = Mean Temperature of Coldest Quarter. bio12 = Annual Precipitation. bio15 = Precipitation Seasonality (Coefficient of Variation). bio16 = Precipitation of Wettest Quarter. bio17 = Precipitation of Driest Quarter. bio18 = Precipitation of Warmest Quarter. bio19 = Precipitation of Coldest Quarter.

| **Department** | **ID** | **Elevation (m)** | **Slope (%)** | **Bioclimatic variables** | | | | | | | | | | | |
| --- | --- | --- | --- | --- | --- | --- | --- | --- | --- | --- | --- | --- | --- | --- | --- |
|  |  |  |  | **bio1** | **bio4** | **bio8** | **bio9** | **bio10** | **bio11** | **bio12** | **bio15** | **bio16** | **bio17** | **bio18** | **bio19** |
| Cerro Largo | 1 | 62.8 | 0.9 | 18.2 | 436.5 | 13.8 | 22.4 | 23.5 | 12.9 | 1316 | 11.0 | 355 | 293 | 326 | 353 |
| Cerro Largo | 2 | 124.0 | 13.9 | 17.9 | 438.1 | 13.4 | 22.1 | 23.2 | 12.5 | 1322 | 10.7 | 356 | 297 | 330 | 352 |
| Cerro Largo | 3 | 159.8 | 1.1 | 17.8 | 437.8 | 13.3 | 22.0 | 23.1 | 12.4 | 1326 | 10.4 | 356 | 298 | 329 | 352 |
| Cerro Largo | 4 | 68.0 | 3.0 | 17.8 | 437.4 | 13.3 | 19.8 | 23.1 | 12.4 | 1294 | 10.9 | 348 | 292 | 326 | 342 |
| Treinta y Tres | 5 | 40.0 | 14.5 | 17.5 | 446.0 | 12.6 | 19.5 | 22.9 | 12.0 | 1283 | 11.0 | 339 | 293 | 330 | 338 |
| Treinta y Tres | 6 | 4.6 | 0.5 | 17.2 | 429.1 | 12.0 | 19.0 | 22.4 | 12.0 | 1266 | 12.2 | 342 | 286 | 335 | 342 |
| Cerro Largo | 7 | 104.3 | 1.8 | 17.4 | 464.8 | 20.7 | 19.7 | 23.0 | 11.7 | 1246 | 6.2 | 324 | 295 | 306 | 318 |
| Tacuarembó | 8 | 98.5 | 8.0 | 18.1 | 462.9 | 21.4 | 20.6 | 23.8 | 12.5 | 1296 | 8.2 | 337 | 310 | 318 | 321 |
| Tacuarembó | 9 | 95.2 | 0.1 | 18.1 | 463.4 | 21.3 | 20.5 | 23.7 | 12.4 | 1280 | 7.9 | 332 | 308 | 312 | 318 |
| Lavalleja | 10 | 85.3 | 2.1 | 16.7 | 447.3 | 11.2 | 18.7 | 22.1 | 11.2 | 1299 | 6.2 | 338 | 303 | 332 | 338 |
| Durazno | 11 | 113.3 | 2.0 | 17.2 | 470.8 | 20.6 | 19.6 | 23.0 | 11.5 | 1226 | 6.9 | 321 | 290 | 298 | 313 |
| Tacuarembo | 12 | 121.0 | 3.3 | 18.2 | 464.9 | 23.5 | 15.1 | 23.8 | 12.6 | 1307 | 11.0 | 353 | 301 | 325 | 304 |
| Tacuarembo | 13 | 131.5 | 1.3 | 18.4 | 459.3 | 23.6 | 12.9 | 24.0 | 12.9 | 1381 | 11.0 | 384 | 308 | 361 | 308 |
| Rocha | 14 | 19.7 | 0.5 | 16.4 | 419.2 | 11.4 | 18.0 | 21.5 | 11.4 | 1203 | 9.8 | 324 | 273 | 305 | 324 |
| Rivera | 15 | 165.1 | 0.7 | 18.3 | 445.3 | 23.4 | 12.9 | 23.7 | 12.9 | 1458 | 11.9 | 413 | 311 | 394 | 311 |
| Lavalleja | 16 | 176.0 | 4.1 | 15.9 | 439.7 | 13.4 | 20.0 | 21.2 | 10.5 | 1277 | 7.1 | 343 | 289 | 296 | 328 |
| Rocha | 17 | 67.7 | 5.6 | 16.0 | 416.8 | 11.0 | 19.8 | 21.1 | 11.0 | 1165 | 7.6 | 311 | 264 | 275 | 311 |
| Florida | 18 | 83.8 | 0.1 | 17.1 | 487.2 | 22.7 | 16.7 | 23.1 | 11.2 | 1256 | 8.0 | 348 | 300 | 326 | 301 |
| Rio Negro | 19 | 126.8 | 1.1 | 17.8 | 489.8 | 23.4 | 11.8 | 23.8 | 11.8 | 1274 | 13.8 | 368 | 268 | 343 | 268 |
| Florida | 20 | 69.1 | 6.7 | 16.6 | 461.1 | 17.2 | 21.0 | 22.2 | 10.9 | 1240 | 7.7 | 333 | 286 | 289 | 306 |
| Lavalleja | 21 | 34.6 | 0.5 | 16.4 | 443.1 | 17.2 | 21.8 | 21.8 | 11.0 | 1170 | 8.9 | 314 | 264 | 264 | 289 |
| Flores | 22 | 40.0 | 0.6 | 17.8 | 493.8 | 23.4 | 11.8 | 23.9 | 11.8 | 1278 | 14.0 | 384 | 282 | 358 | 282 |
| Florida | 23 | 36.3 | 2.7 | 16.8 | 479.5 | 17.3 | 11.0 | 22.7 | 11.0 | 1189 | 8.7 | 324 | 279 | 288 | 279 |
| Paysandu | 24 | 38.9 | 0.3 | 18.3 | 479.9 | 23.8 | 12.4 | 24.2 | 12.4 | 1281 | 19.1 | 390 | 245 | 356 | 245 |
| Paysandú | 25 | 76.1 | 4.9 | 18.1 | 483.3 | 23.7 | 12.2 | 24.0 | 12.2 | 1275 | 18.5 | 390 | 249 | 353 | 249 |
| Paysandu | 26 | 101.1 | 0.2 | 18.3 | 479.0 | 23.8 | 12.4 | 24.1 | 12.4 | 1300 | 21.3 | 399 | 230 | 368 | 230 |
| Soriano | 27 | 25.3 | 51.8 | 18.0 | 490.3 | 23.6 | 12.0 | 24.0 | 12.0 | 1221 | 17.6 | 378 | 248 | 344 | 248 |
| Rio Negro | 28 | 16.9 | 5.2 | 18.1 | 492.0 | 23.7 | 12.1 | 24.1 | 12.1 | 1218 | 18.4 | 379 | 246 | 342 | 246 |
| Paysandú | 29 | 50.1 | 1.2 | 18.4 | 479.4 | 23.9 | 12.5 | 24.3 | 12.5 | 1295 | 23.0 | 400 | 220 | 371 | 220 |
| Rio Negro | 30 | 25.8 | 3.5 | 18.0 | 490.5 | 23.6 | 12.0 | 24.0 | 12.0 | 1155 | 21.4 | 359 | 218 | 322 | 218 |
| Soriano | 31 | 10.6 | 1.5 | 17.7 | 497.7 | 23.3 | 11.6 | 23.8 | 11.6 | 1143 | 22.7 | 368 | 203 | 342 | 203 |
| Paysandú | 32 | 24.3 | 5.1 | 18.2 | 482.4 | 23.9 | 12.2 | 24.1 | 12.2 | 1170 | 24.7 | 354 | 208 | 319 | 208 |

**Table S6.** Mantel test for Pearson correlation between community dissimilarity and environmental distance matrices. p-value in bold indicate significance with p < 0.05.

| Variable | All woody species | | Adults | | Juveniles | |
| --- | --- | --- | --- | --- | --- | --- |
|  | Pearson | *p* | Pearson | *p* | Pearson | *p* |
| Longitude (UTM) | **0.33** | **0.0001** | **0.27** | **0.0002** | **0.24** | **0.0002** |
| Latitude (UTM) | 0.05 | 0.2583 | 0.04 | 0.3159 | 0.11 | 0.0830 |
| Geographic distance | **0.31** | **0.0001** | **0.25** | **0.0001** | **0.29** | **0.0001** |
| Meter above sea level | 0.03 | 0.3026 | 0.01 | 0.3893 | 0.06 | 0.2061 |
| Slope (%) | 0.00 | 0.4425 | -0.06 | 0.7026 | 0.01 | 0.4481 |
| Topographic distance | 0.02 | 0.4104 | -0.06 | 0.6894 | 0.04 | 0.3740 |
| Annual Mean Temperature (a) | 0.04 | 0.3000 | -0.01 | 0.5139 | 0.07 | 0.1979 |
| Temperature Seasonality (b) | **0.13** | **0.0247** | 0.05 | 0.2255 | 0.06 | 0.1910 |
| Mean Temperature of Wettest Quarter | **0.15** | **0.0117** | 0.06 | 0.1391 | 0.07 | 0.1224 |
| Mean Temperature of Driest Quarter | **0.28** | **0.0001** | **0.19** | **0.0004** | **0.21** | **0.0002** |
| Mean Temperature of Warmest Quarter | 0.06 | 0.2328 | -0.01 | 0.5240 | 0.05 | 0.2739 |
| Mean Temperature of Coldest Quarter | 0.06 | 0.1939 | 0.04 | 0.2759 | **0.13** | **0.0486** |
| Climatic distance (a,b) | 0.13 | 0.0510 | 0.04 | 0.3022 | 0.10 | 0.0894 |
| Climatic distance (all temperature variables) | **0.18** | **0.0071** | 0.08 | 0.1287 | **0.15** | **0.0234** |
| Annual Precipitation (c) | 0.07 | 0.2084 | 0.11 | 0.1215 | 0.10 | 0.1571 |
| Precipitation Seasonality (d) | **0.27** | **0.0013** | **0.29** | **0.0007** | **0.23** | **0.0022** |
| Precipitation of Wettest Quarter | **0.17** | **0.0167** | 0.08 | 0.1489 | **0.17** | **0.0191** |
| Precipitation of Driest Quarter | **0.24** | **0.0040** | **0.31** | **0.0007** | **0.21** | **0.0095** |
| Precipitation of Warmest Quarter | 0.15 | 0.0510 | 0.06 | 0.2393 | **0.19** | **0.0215** |
| Precipitation of Coldest Quarter | **0.35** | **0.0001** | **0.34** | **0.0002** | **0.27** | **0.0003** |
| Climatic distance (c,d) | **0.24** | **0.0050** | **0.27** | **0.0028** | **0.24** | **0.0058** |
| Climatic distance (all precipitation variables) | **0.31** | **0.0001** | **0.30** | **0.0007** | **0.30** | **0.0004** |
| Climatic distance (a,b,c,d) | **0.24** | **0.0012** | **0.20** | **0.0052** | **0.22** | **0.0029** |
| Climatic distance (all variables) | **0.30** | **0.0001** | **0.24** | **0.0007** | **0.27** | **0.0001** |

**Table S7.** Partial-mantel test for Pearson correlations between community dissimilarity and environmental distance matrices controlling with geographic distance between plots. p-value in bold indicate significance with p < 0.05.

| Variable | All woody species | | Adults | | Juveniles | |
| --- | --- | --- | --- | --- | --- | --- |
|  | Pearson | *p* | Pearson | *p* | Pearson | *p* |
| Meter above sea level | -0.02 | 0.5972 | -0.03 | 0.6374 | 0.01 | 0.4254 |
| Slope (%) | 0.02 | 0.3641 | -0.05 | 0.6382 | 0.03 | 0.3892 |
| Topographic distance | -0.001 | 0.4736 | -0.07 | 0.7485 | 0.02 | 0.4248 |
| Annual Mean Temperature (a) | -0.19 | 0.9939 | -0.19 | 0.9949 | -0.13 | 0.9466 |
| Temperature Seasonality (b) | -0.06 | 0.8091 | -0.12 | 0.9647 | -0.13 | 0.9470 |
| Mean Temperature of Wettest Quarter | -0.15 | 0.9944 | -0.15 | 0.9944 | -0.15 | 0.9951 |
| Mean Temperature of Driest Quarter | **0.14** | **0.0059** | 0.08 | 0.0747 | 0.06 | 0.1151 |
| Mean Temperature of Warmest Quarter | -0.18 | 0.9907 | -0.23 | 0.9986 | -0.19 | 0.9834 |
| Mean Temperature of Coldest Quarter | -0.13 | 0.9600 | -0.11 | 0.9290 | -0.04 | 0.6609 |
| Climatic distance (a,b) | -0.21 | 0.9936 | -0.28 | 0.9995 | -0.23 | 0.9948 |
| Climatic distance (all temperature variables) | -0.15 | 0.9699 | -0.24 | 0.9991 | -0.19 | 0.9878 |
| Annual Precipitation (c) | -0.05 | 0.7239 | 0.02 | 0.3914 | -0.02 | 0.5544 |
| Precipitation Seasonality (d) | **0.17** | **0.0298** | **0.21** | **0.0119** | 0.13 | 0.0719 |
| Precipitation of Wettest Quarter | 0.04 | 0.2857 | -0.03 | 0.6197 | 0.05 | 0.2670 |
| Precipitation of Driest Quarter | 0.14 | 0.0607 | **0.23** | **0.0091** | 0.11 | 0.1169 |
| Precipitation of Warmest Quarter | 0.03 | 0.3667 | -0.05 | 0.6940 | 0.08 | 0.1825 |
| Precipitation of Coldest Quarter | **0.22** | **0.0061** | **0.25** | **0.0034** | 0.14 | 0.0514 |
| Climatic distance (c,d) | 0.08 | 0.1950 | **0.16** | **0.0494** | 0.10 | 0.1681 |
| Climatic distance (all precipitation variables) | **0.15** | **0.0435** | **0.19** | **0.0229** | 0.15 | 0.0515 |
| Climatic distance (a,b,c,d) | -0.037 | 0.6583 | -0.0001 | 0.4932 | -0.04 | 0.6738 |
| Climatic distance (all variables) | 0.06 | 0.2059 | 0.05 | 0.2751 | 0.03 | 0.3491 |

**Table S8.** Mantel test for Pearson correlation between community dissimilarity and landscape metrics distance matrices. p-value in bold indicate significance with p < 0.05.

| Level | Indices | All | | Adult species | | Juvenile species | |
| --- | --- | --- | --- | --- | --- | --- | --- |
|  |  | Pearson | *p* | Pearson | *p* | Pearson | *p* |
| Landscape | Number of patches | **0.17** | **0.0429** | 0.09 | 0.1585 | **0.17** | **0.0399** |
|  | Landscape shape index | 0.11 | 0.0754 | **0.14** | **0.0438** | 0.10 | 0.1084 |
|  | Shannon's evenness index | 0.09 | 0.1460 | 0.10 | 0.1358 | 0.07 | 0.2120 |
|  | Aggregation index | 0.11 | 0.0667 | **0.14** | **0.0372** | 0.10 | 0.1011 |
|  | Total landscape distance | **0.16** | **0.0319** | **0.15** | **0.0476** | 0.14 | 0.0534 |
|  | Percentage of cover by native forest | -0.05 | 0.6958 | -0.08 | 0.7841 | -0.05 | 0.6630 |
|  | Percentage of cover by natural grassland | 0.05 | 0.2426 | 0.03 | 0.3120 | 0.70 | 0.2849 |
|  | Percentage of cover by crops | 0.12 | 0.1172 | 0.14 | 0.1077 | **0.19** | **0.0463** |
|  | Percentage of cover by timber plantation | **0.25** | **0.0048** | **0.23** | **0.0093** | **0.24** | **0.0072** |
|  | Percentage of cover by sum of timber plantation and crops | **0.29** | **0.0004** | **0.30** | **0.0011** | **0.27** | **0.0016** |
|  | Number of native forest patches | **0.20** | **0.0190** | **0.17** | **0.0447** | 0.16 | 0.0506 |
|  | Interspersion and juxtaposition index for native forest patches | 0.00 | 0.4918 | 0.00 | 0.4869 | 0.02 | 0.4020 |
|  | Mean Euclidean nearest neighbor distance of native forest patches | 0.01 | 0.4046 | 0.00 | 0.4673 | 0.04 | 0.3214 |
|  | Total class distance | **0.27** | **0.0008** | **0.23** | **0.0056** | **0.28** | **0.0009** |
| Patch | Total Area | -0.05 | 0.6739 | -0.09 | 0.7993 | -0.06 | 0.6717 |
|  | Perimeter-Area Ratio | 0.11 | 0.1202 | 0.09 | 0.1823 | 0.12 | 0.1455 |
|  | Shape Index | 0.12 | 0.0745 | 0.07 | 0.2122 | 0.13 | 0.0664 |
|  | Total patch distance | 0.07 | 0.2119 | 0.02 | 0.4167 | 0.08 | 0.2075 |
|  | All landscape metrics (landscape, class and patch metrics) | **0.26** | **0.0008** | **0.22** | **0.0085** | **0.27** | **0.0007** |

**Table S9.** Partial-mantel test for Pearson correlations between community dissimilarity and landscape metrics distance matrices controlling with geographic distance between plots. p-value in bold indicate significance with p < 0.05.

| Level | Indices | All | | Adult species | | Juvenile species | |
| --- | --- | --- | --- | --- | --- | --- | --- |
|  |  | Mantel | *p* | Mantel | *p* | Mantel | *p* |
| Landscape | Number of patches | 0.15 | 0.0648 | 0.07 | 0.2111 | 0.15 | 0.0610 |
|  | Landscape shape index | 0.05 | 0.2211 | 0.09 | 0.1114 | 0.04 | 0.2963 |
|  | Shannon's evenness index | 0.05 | 0.2818 | 0.07 | 0.2159 | 0.03 | 0.3732 |
|  | Aggregation index | 0.06 | 0.2099 | 0.10 | 0.1018 | 0.04 | 0.2806 |
|  | Total landscape distance | 0.10 | 0.1053 | 0.10 | 0.1155 | 0.08 | 0.1565 |
|  | Percentage of cover by native forest | -0.03 | 0.6040 | -0.07 | 0.7159 | -0.03 | 0.5709 |
|  | Percentage of cover by natural grassland | 0.02 | 0.3808 | 0.01 | 0.4271 | 0.01 | 0.4267 |
|  | Percentage of cover by crops | 0.08 | 0.1883 | 0.11 | 0.1567 | 0.16 | 0.0764 |
|  | Percentage of cover by timber plantation | **0.22** | **0.0097** | **0.21** | **0.0140** | **0.21** | **0.0136** |
|  | Percentage of cover by sum of timber plantation and crops | **0.24** | **0.0043** | **0.26** | **0.0039** | **0.22** | **0.0068** |
|  | Number of native forest patches | **0.25** | **0.0059** | **0.20** | **0.0244** | **0.21** | **0.0201** |
|  | Interspersion and juxtaposition index for native forest patches | -0.06 | 0.7406 | -0.04 | 0.6748 | -0.03 | 0.6253 |
|  | Mean Euclidean nearest neighbor distance of native forest patches | 0.03 | 0.3415 | 0.01 | 0.4145 | 0.05 | 0.2659 |
|  | Total class distance | **0.24** | **0.0026** | **0.20** | **0.0108** | **0.25** | **0.0027** |
| Patch | Total Area | -0.03 | 0.5860 | -0.08 | 0.7499 | -0.03 | 0.5988 |
|  | Perimeter-Area Ratio | 0.13 | 0.1030 | 0.10 | 0.1631 | 0.13 | 0.1161 |
|  | Shape Index | 0.12 | 0.0732 | 0.06 | 0.2121 | 0.13 | 0.0702 |
|  | Total patch distance | 0.11 | 0.1386 | 0.04 | 0.3360 | 0.11 | 0.1353 |
|  | All landscape metrics (landscape, class and patch metrics) | **0.23** | **0.0036** | **0.18** | **0.0224** | **0.23** | **0.0039** |

**Table S10.** Linear distance matrix between permanent plots (or sites) in kilometer Km (below of diagonal) and Jaccard index (above of diagonal). Number represent each forest fragments. The matrix was ordered according linear distance between 1 and 32 sites (see column 1) and Fig. 1 from main text.

| **ID** | **1** | **2** | **3** | **4** | **5** | **6** | **7** | **8** | **9** | **10** | **11** | **12** | **13** | **14** | **15** | **16** | **17** | **18** | **19** | **20** | **21** | **22** | **23** | **24** | **25** | **26** | **27** | **28** | **29** | **30** | **31** | **32** |
| --- | --- | --- | --- | --- | --- | --- | --- | --- | --- | --- | --- | --- | --- | --- | --- | --- | --- | --- | --- | --- | --- | --- | --- | --- | --- | --- | --- | --- | --- | --- | --- | --- |
| **1** |  | 0.543 | 0.214 | 0.318 | 0.512 | 0.487 | 0.237 | 0.385 | 0.342 | 0.342 | 0.250 | 0.243 | 0.167 | 0.378 | 0.282 | 0.128 | 0.306 | 0.282 | 0.225 | 0.184 | 0.250 | 0.108 | 0.184 | 0.182 | 0.195 | 0.098 | 0.205 | 0.250 | 0.125 | 0.132 | 0.216 | 0.093 |
| **2** | 4 |  | 0.317 | 0.356 | 0.511 | 0.356 | 0.220 | 0.295 | 0.286 | 0.317 | 0.262 | 0.225 | 0.156 | 0.317 | 0.262 | 0.175 | 0.316 | 0.178 | 0.182 | 0.171 | 0.205 | 0.073 | 0.143 | 0.196 | 0.182 | 0.067 | 0.163 | 0.200 | 0.091 | 0.122 | 0.171 | 0.111 |
| **3** | 7 | 3 |  | 0.367 | 0.263 | 0.281 | 0.429 | 0.370 | 0.360 | 0.308 | 0.435 | 0.318 | 0.185 | 0.308 | 0.222 | 0.286 | 0.364 | 0.179 | 0.280 | 0.217 | 0.179 | 0.143 | 0.217 | 0.296 | 0.231 | 0.077 | 0.200 | 0.273 | 0.167 | 0.182 | 0.217 | 0.200 |
| **4** | 23 | 21 | 23 |  | 0.341 | 0.263 | 0.321 | 0.333 | 0.323 | 0.323 | 0.290 | 0.333 | 0.258 | 0.242 | 0.333 | 0.133 | 0.233 | 0.143 | 0.182 | 0.129 | 0.176 | 0.148 | 0.167 | 0.235 | 0.182 | 0.129 | 0.156 | 0.296 | 0.129 | 0.138 | 0.250 | 0.156 |
| **5** | 64 | 62 | 62 | 42 |  | 0.447 | 0.333 | 0.342 | 0.297 | 0.371 | 0.237 | 0.303 | 0.211 | 0.333 | 0.270 | 0.171 | 0.294 | 0.205 | 0.243 | 0.235 | 0.237 | 0.152 | 0.135 | 0.195 | 0.243 | 0.105 | 0.158 | 0.273 | 0.167 | 0.143 | 0.235 | 0.128 |
| **6** | 114 | 114 | 115 | 92 | 60 |  | 0.321 | 0.467 | 0.414 | 0.367 | 0.250 | 0.286 | 0.219 | 0.414 | 0.290 | 0.214 | 0.370 | 0.250 | 0.182 | 0.207 | 0.212 | 0.148 | 0.207 | 0.200 | 0.258 | 0.094 | 0.233 | 0.296 | 0.129 | 0.138 | 0.296 | 0.156 |
| **7** | 151 | 147 | 144 | 138 | 112 | 145 |  | 0.320 | 0.304 | 0.304 | 0.381 | 0.471 | 0.333 | 0.250 | 0.381 | 0.211 | 0.300 | 0.160 | 0.333 | 0.200 | 0.208 | 0.176 | 0.143 | 0.292 | 0.333 | 0.091 | 0.130 | 0.412 | 0.143 | 0.100 | 0.333 | 0.182 |
| **8** | 151 | 147 | 143 | 148 | 143 | 194 | 74 |  | 0.762 | 0.321 | 0.333 | 0.391 | 0.167 | 0.423 | 0.241 | 0.250 | 0.269 | 0.286 | 0.296 | 0.192 | 0.241 | 0.174 | 0.240 | 0.407 | 0.296 | 0.069 | 0.222 | 0.409 | 0.148 | 0.115 | 0.292 | 0.138 |
| **9** | 159 | 154 | 151 | 155 | 146 | 196 | 68 | 11 |  | 0.360 | 0.269 | 0.450 | 0.185 | 0.417 | 0.222 | 0.227 | 0.250 | 0.320 | 0.280 | 0.217 | 0.269 | 0.200 | 0.273 | 0.346 | 0.280 | 0.077 | 0.250 | 0.474 | 0.120 | 0.130 | 0.333 | 0.111 |
| **10** | 180 | 178 | 177 | 159 | 117 | 98 | 104 | 176 | 172 |  | 0.320 | 0.381 | 0.231 | 0.619 | 0.222 | 0.227 | 0.429 | 0.269 | 0.333 | 0.273 | 0.435 | 0.263 | 0.273 | 0.296 | 0.280 | 0.167 | 0.250 | 0.333 | 0.273 | 0.238 | 0.400 | 0.250 |
| **11** | 184 | 180 | 178 | 169 | 138 | 160 | 37 | 102 | 94 | 95 |  | 0.273 | 0.192 | 0.269 | 0.280 | 0.300 | 0.450 | 0.185 | 0.348 | 0.125 | 0.280 | 0.095 | 0.125 | 0.214 | 0.192 | 0.080 | 0.115 | 0.227 | 0.125 | 0.136 | 0.174 | 0.208 |
| **12** | 210 | 206 | 202 | 210 | 207 | 258 | 127 | 64 | 62 | 231 | 143 |  | 0.350 | 0.381 | 0.474 | 0.222 | 0.250 | 0.273 | 0.350 | 0.278 | 0.333 | 0.357 | 0.278 | 0.429 | 0.421 | 0.095 | 0.316 | 0.769 | 0.211 | 0.167 | 0.533 | 0.136 |
| **13** | 218 | 214 | 211 | 221 | 224 | 279 | 156 | 86 | 88 | 260 | 176 | 34 |  | 0.185 | 0.409 | 0.190 | 0.217 | 0.107 | 0.200 | 0.182 | 0.192 | 0.294 | 0.130 | 0.222 | 0.200 | 0.182 | 0.167 | 0.368 | 0.130 | 0.143 | 0.368 | 0.217 |
| **14** | 221 | 220 | 221 | 199 | 162 | 107 | 201 | 268 | 266 | 101 | 195 | 327 | 354 |  | 0.222 | 0.286 | 0.364 | 0.320 | 0.455 | 0.400 | 0.435 | 0.333 | 0.333 | 0.400 | 0.333 | 0.077 | 0.364 | 0.400 | 0.273 | 0.238 | 0.333 | 0.154 |
| **15** | 225 | 222 | 219 | 232 | 241 | 298 | 184 | 111 | 116 | 288 | 207 | 68 | 34 | 379 |  | 0.130 | 0.261 | 0.231 | 0.240 | 0.227 | 0.280 | 0.211 | 0.227 | 0.214 | 0.292 | 0.174 | 0.261 | 0.421 | 0.174 | 0.190 | 0.421 | 0.208 |
| **16** | 251 | 249 | 248 | 229 | 187 | 158 | 161 | 234 | 228 | 71 | 137 | 280 | 312 | 104 | 343 |  | 0.278 | 0.182 | 0.389 | 0.313 | 0.238 | 0.133 | 0.235 | 0.273 | 0.250 | 0.050 | 0.278 | 0.235 | 0.235 | 0.267 | 0.167 | 0.211 |
| **17** | 272 | 271 | 271 | 250 | 210 | 163 | 216 | 289 | 284 | 113 | 199 | 341 | 371 | 66 | 400 | 70 |  | 0.208 | 0.217 | 0.263 | 0.261 | 0.111 | 0.200 | 0.148 | 0.217 | 0.091 | 0.238 | 0.200 | 0.143 | 0.222 | 0.263 | 0.238 |
| **18** | 277 | 274 | 272 | 260 | 224 | 227 | 134 | 190 | 180 | 132 | 97 | 211 | 245 | 216 | 279 | 120 | 189 |  | 0.240 | 0.421 | 0.333 | 0.150 | 0.350 | 0.214 | 0.192 | 0.080 | 0.318 | 0.350 | 0.174 | 0.250 | 0.286 | 0.160 |
| **19** | 283 | 278 | 275 | 274 | 254 | 286 | 143 | 140 | 129 | 219 | 128 | 116 | 144 | 319 | 177 | 241 | 310 | 134 |  | 0.300 | 0.409 | 0.294 | 0.182 | 0.375 | 0.250 | 0.083 | 0.217 | 0.368 | 0.238 | 0.143 | 0.238 | 0.167 |
| **20** | 283 | 281 | 280 | 262 | 221 | 200 | 170 | 241 | 233 | 105 | 139 | 278 | 312 | 151 | 344 | 48 | 108 | 87 | 219 |  | 0.286 | 0.286 | 0.375 | 0.261 | 0.300 | 0.100 | 0.412 | 0.375 | 0.375 | 0.333 | 0.375 | 0.200 |
| **21** | 310 | 308 | 308 | 288 | 247 | 216 | 211 | 283 | 276 | 130 | 182 | 323 | 356 | 144 | 388 | 60 | 85 | 131 | 264 | 46 |  | 0.150 | 0.227 | 0.259 | 0.292 | 0.125 | 0.261 | 0.350 | 0.174 | 0.190 | 0.350 | 0.261 |
| **22** | 317 | 313 | 311 | 304 | 273 | 288 | 166 | 197 | 186 | 201 | 135 | 193 | 225 | 291 | 259 | 196 | 265 | 76 | 86 | 161 | 202 |  | 0.200 | 0.250 | 0.158 | 0.059 | 0.250 | 0.385 | 0.286 | 0.143 | 0.286 | 0.111 |
| **23** | 335 | 332 | 330 | 315 | 275 | 262 | 204 | 266 | 257 | 164 | 168 | 290 | 324 | 217 | 358 | 114 | 168 | 79 | 207 | 66 | 86 | 130 |  | 0.261 | 0.300 | 0.100 | 0.600 | 0.294 | 0.375 | 0.333 | 0.375 | 0.200 |
| **24** | 349 | 345 | 342 | 343 | 325 | 359 | 215 | 200 | 191 | 290 | 201 | 158 | 177 | 389 | 203 | 306 | 375 | 191 | 73 | 278 | 322 | 122 | 252 |  | 0.375 | 0.115 | 0.240 | 0.381 | 0.261 | 0.227 | 0.381 | 0.192 |
| **25** | 349 | 345 | 342 | 342 | 322 | 353 | 211 | 203 | 193 | 281 | 194 | 165 | 186 | 379 | 215 | 293 | 363 | 177 | 68 | 264 | 307 | 106 | 236 | 18 |  | 0.083 | 0.273 | 0.368 | 0.300 | 0.200 | 0.444 | 0.167 |
| **26** | 357 | 353 | 350 | 353 | 339 | 377 | 232 | 206 | 198 | 315 | 222 | 155 | 168 | 415 | 190 | 335 | 404 | 223 | 96 | 310 | 354 | 156 | 287 | 35 | 53 |  | 0.091 | 0.100 | 0.158 | 0.176 | 0.222 | 0.200 |
| **27** | 364 | 360 | 358 | 352 | 324 | 342 | 215 | 234 | 222 | 255 | 186 | 216 | 244 | 345 | 277 | 248 | 316 | 129 | 101 | 210 | 248 | 54 | 168 | 100 | 82 | 134 |  | 0.412 | 0.333 | 0.294 | 0.333 | 0.182 |
| **28** | 371 | 366 | 364 | 359 | 332 | 351 | 222 | 237 | 226 | 266 | 194 | 216 | 243 | 356 | 275 | 260 | 328 | 141 | 101 | 222 | 261 | 65 | 181 | 91 | 73 | 125 | 13 |  | 0.222 | 0.176 | 0.571 | 0.143 |
| **29** | 376 | 372 | 368 | 372 | 358 | 397 | 252 | 225 | 217 | 334 | 242 | 172 | 182 | 434 | 203 | 353 | 423 | 239 | 115 | 327 | 371 | 171 | 301 | 49 | 65 | 20 | 144 | 134 |  | 0.333 | 0.294 | 0.143 |
| **30** | 405 | 401 | 398 | 395 | 371 | 394 | 259 | 265 | 254 | 311 | 235 | 234 | 257 | 402 | 286 | 306 | 375 | 187 | 125 | 268 | 306 | 111 | 225 | 84 | 71 | 111 | 59 | 46 | 113 |  | 0.333 | 0.294 |
| **31** | 413 | 409 | 407 | 401 | 372 | 387 | 263 | 282 | 271 | 297 | 233 | 262 | 289 | 382 | 320 | 281 | 347 | 166 | 147 | 239 | 273 | 99 | 188 | 128 | 112 | 159 | 49 | 46 | 163 | 52 |  | 0.333 |
| **32** | 414 | 410 | 407 | 406 | 386 | 415 | 275 | 267 | 257 | 339 | 255 | 226 | 243 | 434 | 268 | 342 | 412 | 223 | 132 | 308 | 348 | 147 | 269 | 68 | 65 | 80 | 101 | 88 | 75 | 49 | 100 |  |

1. Jaccard, P. (1912), The distribution of the flora in the alpine zone. New Phytologist, 11: 37-50. <https://doi.org/10.1111/j.1469-8137.1912.tb05611.x>. All other sources are cited in the main text. [↑](#footnote-ref-1)
